# Trends and developments in health systems modeling: a bibliometric analysis

**DOI:** 10.3389/fdgth.2025.1595310

**Published:** 2025-10-14

**Authors:** Kudakwashe Maguraushe, Patrick Ndayizigamiye, Tebogo Bokaba

**Affiliations:** ^1^ Department of Applied Information Systems, University of Johannesburg, Johannesburg, South Africa; ^2^ Centre for Applied Data Science, University of Johannesburg, Johannesburg, South Africa

**Keywords:** health systems modeling, bibliometric analysis, science mapping, performance analysis, predictive modeling

## Abstract

**Introduction:**

Health systems modeling is increasingly used to address complex health challenges and inform policy. Despite its growing importance, the field remains dynamic, with evolving research themes, and global contributions. This study maps the evolution of the field, identifies leading publications, authors, institutions, and countries, and highlights emerging themes to guide future research and collaboration.

**Methods:**

A bibliometric analysis was conducted on March 10, 2023, using the Web of Science (WoS) Core Collection for 1992–2023. The search string was “health system*” AND “modelling” OR “modeling.” Records were analyzed with Biblioshiny and VOSviewer to compute publication trends, authorship patterns, institutional and country-level contributions, international collaboration, and thematic developments.

**Results:**

A total of 2,023 records were retrieved. The annual publication growth rate was 7.53%, with an average of 9.35 co-authors per article and 37.67% international co-authorship. Leading journals included The Lancet and PLOS One, while prominent authors were Blakely T. and Hay S.I. Key contributing institutions were the Tehran University of Medical Sciences and the University of Washington. The United States and the United Kingdom were the most productive countries. Thematic analysis revealed prominent and emerging topics such as “health systems,” “modeling,” “predictive modeling,” and “systems dynamics” suggesting promising directions for future research.

**Discussion:**

Findings indicate a dynamic and expanding research landscape with strong international collaboration and concentrated contributions from high-impact journals, established authors, and leading institutions. The study highlights epidemiology and predictive modeling as promising directions for future research and identifies opportunities for international collaboration and publication. The analysis is limited by reliance on a single database (WoS); further studies should integrate additional databases to improve coverage and deepen the findings. The results can inform decisions on collaboration opportunities, suitable publication venues, and key research gaps in health systems modeling.

## 1 Introduction

Accurate modeling techniques have necessitated protracted efforts to predict trends and developments in health systems (1). The concept of modeling in health systems has been prevalent, as many life-threatening diseases can be predicted based on the available data (2). Globally, there has been an increasing trend in utilizing modeling applications in the healthcare industry to address complex situations (3). It is widely recognized that modeling significantly predicts future outcomes and enhances decision-making processes (1). Healthcare systems can better prepare their responses by predicting potential future epidemics, ultimately reducing the strain on human resources (4). Modeling of health systems has increasingly been applied to enhance decision-making in various health issues that include digital prosthetics (5–7); COVID-19 diagnosis and prognosis (4, 8, 9); contact tracing, household quarantine and future waves of COVID-19 transmission (1, 10, 11); predicting the impact of COVID-19 on service disruption (12, 13); psychological effects of the COVID-19 pandemic (14, 15); COVID-19 transmission and mitigation strategies (10); predicting a model for the adoption of personal health records (3); prediction of cancer death rate (2, 16, 17); effects of density on the spread of contagious diseases (18); detection of magnetic resonance image (MRI) diagnosis (19); modeling tissue development (20); enhancement of data distribution services (DDS) security (21); heart rate estimation (22); cancer screening modeling techniques (16); and prediction of suicide risk (23). A recent case scenario was during the peak of the COVID-19 pandemic, where some lives were saved because several modeling studies pointed out that the resumption of economic activities and social life would reignite the resurgence of the pandemic (11).

The use of modeling techniques in the healthcare industry has experienced a substantial and steady increase, primarily focused on improving decision-making processes and offering a strategic shift towards predicting future outcomes (5, 24). This growing trend is driven by the ever-increasing demand for healthcare services and the critical need for efficient and sustainable healthcare frameworks (25–27). Furthermore, the field of simulation modeling, encompassing discrete-event simulation, system dynamics, agent-based modeling, Monte Carlo simulation, and hybrid systems, marks a significant milestone in healthcare system modeling (28). This evolution has been characterized by a notable shift towards integrating mixed-methods, hybrid, and multi-paradigm methodologies.

Musculoskeletal modeling is a prime example in the clinical domain, finding applications across various clinical settings (29). These range from non-invasive treatments and orthotic assessments to critical surgical decision-making, evaluation of surgical interventions, and the intricate assessment of rehabilitation programmes. Moreover, the integration of artificial intelligence (AI) and machine learning (ML) technologies has revolutionized the precision and computational efficiency of analyses within healthcare (24, 30, 31). These advancements are not merely technical progressions but a profound deepening in the scope and capability of healthcare system modeling to address and navigate complex scenarios, including the advancement of digital prosthetics (5). Furthermore, the fusion of Internet of Things (IoT) technology with ML has demonstrated exceptional effectiveness in health prediction systems. ML algorithms adeptly manage and interpret data generated by IoT devices to predict vital healthcare information (31, 32), showcasing a synergistic relationship between technology and healthcare that could revolutionize predictive healthcare analytics.

Innovative technologies are critically needed in healthcare facilities to recognize symptoms for precise diagnosis and severity forecasting (9). Modeling will remain an important tool for understanding trade-offs in the health system and guiding solid decision-making (12). It is challenging to run efficient, long-lasting healthcare systems because of changing demographics, healthcare funding restrictions, and the rising demand for healthcare services. Healthcare systems modeling can assist in optimizing resource use, considering the complexity of healthcare delivery. Thus, obtaining more insights into health systems modeling is imperative.

Although health systems modeling is increasingly utilized for epidemic forecasting, operational optimization, cost-effectiveness assessments, and digital health transformation, existing literature lacks a unified, wide-scope bibliometric mapping of the field. Many reviews have either focused narrowly on specific modeling methods or isolated domains such as discrete-event simulation in healthcare operations (33), simulation-based medical education (34), artificial intelligence applications (35), or virtual reality in healthcare (36) without capturing the broader intellectual structure, geographic evolution, or interdisciplinary dynamics of health system modeling. Moreover, while bibliometric tools such as CiteSpace, VOSviewer, and Biblioshiny have been successfully used to visualize scientific progress and collaboration networks (37), few studies have integrated both science mapping and performance analysis to uncover the key thematic clusters systematically, most cited authors, and institutional ecosystems shaping the field. To address these gaps, this study applies a comprehensive bibliometric analysis to 2,023 publications on health systems modeling indexed in the Web of Science from 1992–2023. According to (38), bibliometric analysis is a quantitative method used to study trends in scholarly documents such as research articles, conference papers, books, and other publications. By combining performance analysis (e.g., citation counts, author productivity, institutional rankings) with science mapping techniques (e.g., keyword co-occurrence and thematic evolution), the study aims to provide a structured and inclusive overview of the field's historical trajectory, knowledge hubs, key themes and concepts arising in the health systems modeling discourse, and promising future directions.

The remainder of the article is structured as follows: Section 2 presents the methods used, Section 3 presents the results of the bibliometric analysis, Section 4 discusses the results, and Section 5 concludes the article.

## 2 Thematic framework

Given the increasing sophistication of health issues and the necessity for evidence-based decision-making, the discipline of health systems modeling has experienced substantial growth. The growing range of modeling methodologies and applications has resulted in a fragmented body of literature. To improve conceptual clarity and enable the synthesis of prior research, this study presents a two-dimensional framework that categorizes health systems modeling studies according to: (i) their primary objective, i.e., predictive vs. explanatory, and (ii) their modeling approach, i.e., data-driven vs. system-based.

### 2.1 Predictive vs. explanatory—objective of the model

The initial dimension differentiates between predictive and explanatory models. Predictive models seek to anticipate future conditions of a health system under diverse scenarios (39). These are frequently predictive and are frequently employed to forecast outcomes such as illness prevalence, hospital admissions, or resource requirements across various scenarios (40). For instance, during the COVID-19 pandemic, prediction models were essential in assessing the effects of measures such as lockdowns and vaccination tactics (41). These models often emphasize accuracy, generalizability, and practical applicability.

In contrast, explanatory models aim to comprehend the mechanisms and reasons behind specific results. These models are predominantly theory-driven and are employed to investigate the fundamental mechanics of complex systems, including health service utilization behaviors, interactions among system components, or the impacts of policy instruments (42). System dynamics models are frequently created to replicate feedback loops and delays in health systems, providing insights into systemic inefficiencies or unexpected effects of treatments (43).

This distinction elucidates whether a model is designed to facilitate quick decision-making through forecasts or to enhance comprehension of system behavior over time.

### 2.2 Data-driven vs. system-based—modeling approach

The second dimension emphasizes the modeling process. At one end are data-driven models that depend primarily on empirical data and statistical or machine learning approaches (44). These models are frequently employed for disease classification, risk prediction, and patient outcomes forecasting (45). The emergence of big data and the widespread use of electronic health records have elevated data-driven approaches, including deep learning, ensemble models, and clustering, particularly in precision medicine and public health surveillance (46).

On the other end are system-oriented models, including system dynamics, agent-based modeling (ABM), and discrete event simulation (DES) (47). These models typically rely on theoretical frameworks and aim to emulate the dynamic interactions among components within health systems. For instance, ABMs can replicate patient trajectories inside sophisticated healthcare systems, whereas DES can analyze patient movement within hospitals or clinics (48). These approaches are particularly practical for capturing nonlinearities, feedback loops, and temporal delays that define real-world health systems (47).

## 3 Methods

The bibliometric approach has been widely utilised to track and assess research development in fields like Artificial Intelligence (AI) (49, 50), critical thinking in primary education (51), big data (52, 53), smart learning environments (54), and trends in social media (55), among many others. A bibliometric analysis helps establish publication patterns within a selected field based on metrics such as contribution to global scholarship through collaborations, authorship, institutional affiliations, and the country of origin of the authors, among others (38). The bibliometric approach is characterized by its capacity to undertake extensive analysis of a wide-ranging field of research, making it a macro-oriented method. Consequently, researchers utilizing this method are not required to specify the particular relationships they aim to investigate, as noted by (53), which enhances objectivity in the assessment of the relevant literature.

Science mapping and bibliometric performance analysis are the primary techniques used in bibliometric methodologies (56–58). (57) argue that science mapping is a valuable tool for representing and analysing the social and cognitive structures within a scientific field, which are constantly evolving. On the other hand, bibliometric performance analysis employs bibliographic data to evaluate the productivity of individual scholars and entities, including authors, journals, institutions, and countries represented through the authors' affiliations (56). In other words, bibliometric performance analysis helps assess scientific scholars based on their impact on their research. Both techniques help ascertain the trends and developments in health systems modeling.

The study used the bibliometric analysis approach to analyze the characteristics of distinct publications and identify research trends in health systems modeling. The number of scientific articles has significantly increased, making it difficult for researchers to follow through with the literature related to their field of interest (51). In bibliometric analysis, specific articles or documents, such as the author, publication information, subject area, cited author, and referenced sources, are statistically and quantitatively analyzed (57). This study used the Web of Science (WoS) database for the bibliometric analysis. The WoS is renowned for its extensive coverage of high-quality, peer-reviewed journals across various disciplines (59). This comprehensive coverage ensured that our bibliometric analysis encompassed various influential and foundational articles in health systems modeling, thus providing a robust foundation for identifying trends and developments.

The search was done on March 10 2023, using the keywords “health system*” AND (“modelling” OR “modeling”) to identify articles on systems modeling in the healthcare domain. This combination was selected to capture a broad yet relevant literature addressing health systems' structure, processes, and performance using systems science and modelling approaches. The use of the asterisk (*) as a truncation symbol helps capture variations such as “health system” and “health systems”.

No timeline restrictions were imposed in the search process. The results were filtered as follows to yield only journal and conference papers published in English: TITLE-ABS-KEY (“health system*” AND “modelling” OR “modeling”) AND [LIMIT-TO (LANGUAGE, “English”)] AND [LIMIT-TO (SRCTYPE, “j”) OR LIMIT-TO (SRCTYPE, “p”)].

**Figure 1:**
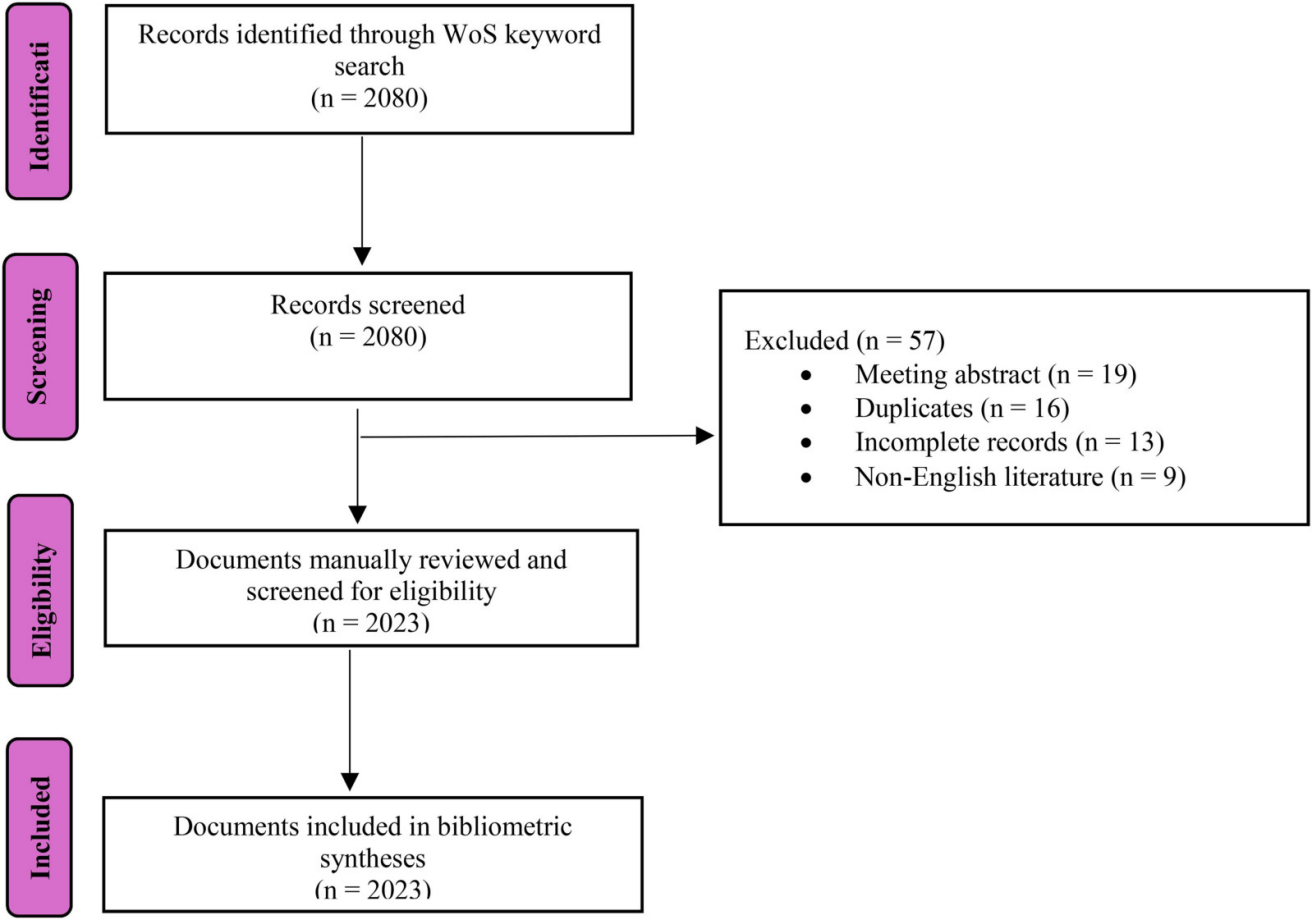
Flow diagram demonstrating data screening, eligibility, and final inclusion processes.

Both Biblioshiny and VOSviewer software were used for the bibliometric analysis and to visualise the results. Both are open-access and free-to-download software, often used to visualize and interpret large bibliometric datasets (60). VOSviewer software helps create networks of journals, scholars, or articles based on citation, bibliographic coupling, co-authorship associations, words used in the publication, and the co-occurrence of keywords within the text (51). On the other hand, Biblioshiny, a statistical software, is used for data mining in bibliometrics to determine the frequency of concurrent keyword occurrences in scientific articles, simplifying the complex keyword network linkages (61). After retrieval, the data were cleaned to address common issues in bibliometric datasets. Duplicates were identified and removed, author names and affiliations were standardized, and incomplete records were either completed or excluded. A flowchart to detail the processes involved is depicted in Figure 1.

Figure 1 systematically outlines the data selection process from initial identification to final inclusion. Initially, 2,080 records were identified, with 57 excluded due to meeting abstracts, duplication, incomplete data, or non-English language. This rigorous screening reduced the dataset to 2,023 relevant documents. The figure delineates each step, enhancing transparency and reproducibility. Notably, the figure demonstrates methodological rigor and transparency in dataset construction, which is critical for bibliometric credibility.

## 4 Results

The search yielded 2,023 records, as shown in Table 1. The records comprised 1,953 journal articles, 36 proceedings articles, and 34 early access articles. The 34 early access articles were included based on their first appearance in the WoS database, as they are publicly accessible and citable despite not being assigned to a final journal issue. This accurately represents current academic output and aligns with bibliometric standards (Clarivate, 2023), reflecting true research trends and achievements. The search results also indicate that scholars within the health system modeling domain are keen on collaborating, with an average of 9.35 co-authors per article and a 37.67% international co-authorship.

**Table 1:** Key information regarding the collection.

Description	Value
Timespan	1992:2023
Sources (journals, books, etc)	931
Documents	2023
Annual growth rate %	7.53
Document average age	5.42
Average citations per doc	22.82
References	72,714
Document contents	
Keywords plus (ID)	4,163
Author's keywords (DE)	5,106
Authors	
Authors	14,386
Authors of single-authored docs	60
Authors collaboration	
Single-authored docs	62
Co-authors per doc	9.35
International co-authorships %	37.67
Document types	
Article	1953
Article, early access	34
Article, proceedings paper	36

### 4.1 Current state of health systems modeling

The scholarly literature on health systems modeling can be traced back to 1992, when it began with a modest corpus of merely two articles. However, recent trends indicate a remarkable surge in scholarly output, with 2021 witnessing the highest output on the subject matter, namely, 351 publications. The period under review (1992–2023) saw an annual growth rate of 7.53%, with a high average citation per document index of 22.82. This is a strong growth rate compared with the approximately 3% annual growth rate in the global scientific publication output (62). Substantial growth in health systems modeling research occurred between 2015 and 2022, with 2023 already having 19 publications. There has been a marked surge in the utilisation of health systems modeling in tackling chronic, tobacco-related, and cancer-related illnesses, as well as other health-related concerns. This trend has been attributed to the recognition that systems modeling can contribute to a more comprehensive understanding of the intricate and complex nature underpinning health and disease prevention (63).

The notable upsurge in publications on health systems modeling between 2020 and 2022 can be attributed to the onset of the COVID-19 pandemic. This global crisis inflicted significant harm on human lives and necessitated the development of precise prediction mechanisms to address its impact on public health (1). Many different co-authorship indices are also revealed. The average number of co-authors for each article is the basis for the co-authors per article index (64). In this study, the index coefficient is 9.35.

Table 2 depicts two documents published in 1992, when articles on health systems modeling were first cited. The first article to be published was by Schinnar, Rothbard & Hadley from the University of Pennsylvania titled “A Prospective Management Approach to the Delivery of Public Mental Health Services” in the journal Administration and Policy in Mental Health and Mental Health Services Research in March 1992. This foundational article contributed to the early conceptual framing of health systems modeling by introducing a system-oriented strategy encompassing forecasting service demands, optimizing resource allocation, and employing decision-support mechanisms within public mental health services. Subsequent literature has cited this work in support of broader system-level planning frameworks. For example (65), was built on (66), emphasizing performance evaluation to apply Data Envelopment Analysis (DEA) in funding allocation for behavioral health contractors. Similarly (67), referenced the study about assessing community mental health service needs and forecasting future demand for planning purposes.

**Table 2:** Annual publications and citations.

Year	MeanTCperArt	N	MeanTCperYear	CitableYears
1992	4.5	2	0.14	32
1994	51.33	3	1.71	30
1995	13.33	3	0.46	29
1996	16.67	3	0.6	28
1997	16.17	6	0.6	27
1998	36.5	2	1.4	26
1999	14.67	6	0.59	25
2000	99.67	9	4.15	24
2001	42.86	7	1.86	23
2002	14	2	0.64	22
2003	23.5	2	1.12	21
2004	36.75	8	1.84	20
2005	162.86	7	8.57	19
2006	39.08	12	2.17	18
2007	47	7	2.76	17
2008	41.17	23	2.57	16
2009	38.32	19	2.55	15
2010	47.56	27	3.4	14
2011	32.82	39	2.52	13
2012	32.89	62	2.74	12
2013	31.28	60	2.84	11
2014	28.82	76	2.88	10
2015	28.8	110	3.2	9
2016	46.59	126	5.82	8
2017	68.74	133	9.82	7
2018	15.58	146	2.6	6
2019	15.68	167	3.14	5
2020	22.11	233	5.53	4
2021	8.34	351	2.78	3
2022	1.95	320	0.98	2
2023	0.32	19	0.32	1

### 4.2 Most influential publications in the field of health systems modeling

The health systems modeling discipline encompasses diverse domains, comprising but not limited to surveillance mechanisms for identifying health threats, interventions aimed at controlling and mitigating the impact of such threats on public health, and predictive technologies designed to address various health-related challenges. The following criteria were used to articulate the most influential publications in health systems modeling: (i) the most cited articles, (ii) publications with the highest number of articles, (iii) publications with the highest number of citations, and (iv) publications with top indexing figures.

#### 4.2.1 Most cited articles and publications

Table 3 shows the top 10 most cited articles and publications, represented by the author(s), the year the article was published, the title of the article, the journal that published the article, and the total number of citations (TC), for the research on health systems modeling. The bibliometric analysis identified the article authored by (68), titled: “Global, regional, and national life expectancy, all-cause mortality, and cause-specific Mortality for 249 Causes of Death, 1980–2015: A Systematic Analysis for the Global Burden of Disease Study 2015”, published by The Lancet as the most cited article worldwide, with the TC = 2,881. Vos T et al., (69) in an article titled: “Global, Regional, and National Incidence, Prevalence, and Years Lived with Disability for 328 Diseases and Injuries for 195 Countries, 1990–2016: A Systematic Analysis for the Global Burden of Disease Study” was the second most cited article, also published by The Lancet and with TC = 2,686. In fact, all the top 10 articles were published in journals with high impact factors, and six of the 10 articles were made up of multiple collaborators within the healthcare domain.

**Table 3:** Top 10 most cited articles and publications.

Rank	Authors/year	Title	Sources	TC
1	Wang HD et al., (68)	Global, regional, and national life expectancy, all-cause mortality, and cause-specific mortality for 249 causes of death, 1980–2015: A systematic analysis for the Global Burden of Disease Study 2015	The Lancet	2,881
2	Vos T et al., (69)	Global, regional, and national incidence, prevalence, and years lived with disability for 328 diseases and injuries for 195 countries, 1990–2016: A systematic analysis for the Global Burden of Disease Study 2016	The Lancet	2,686
3	Roth GA et al., (70)	Global, regional, and national burden of cardiovascular diseases for 10 causes, 1990 to 2015	Journal of the American College of Cardiology	1,895
4	Hay SI et al., (71)	Global, regional, and national disability-adjusted life-years (DALYs) for 333 diseases and injuries and healthy life expectancy (HALE) for 195 countries and territories, 1990–2016: A systematic analysis for the Global Burden of Disease Study 2016	The Lancet	1,878
5	Krieger N et al., (77)	Experiences of discrimination: Validity and reliability of a self-report measure for population health research on racism and health	Social Science & Medicine	976
6	Santomauro DF et al., (74)	Global prevalence and burden of depressive and anxiety disorders in 204 countries and territories in 2020 due to the COVID-19 pandemic	The Lancet	697
7	Sanson-Fisher R et al., (72)	The unmet supportive care needs of patients with cancer	Cancer	633
8	Roberton T et al., (75)	Early estimates of the indirect effects of the COVID-19 pandemic on maternal and child mortality in low-income and middle-income countries: A modeling study	Lancet Global Health	594
9	Piel FB et al., (73)	Global epidemiology of sickle haemoglobin in neonates: A contemporary geostatistical model-based map and population estimates	The Lancet	585
10	Jendritzky G et al., (76)	UTCI—Why another thermal index?	International Journal of Biometeorology	503

The Supplementary Material provides a detailed analysis of the most cited papers, focusing on the paper context, methodologies, findings, conclusions, and suggestions for future research as depicted in the studies. The 10 research papers span various topics across various fields, including oncology, epidemiology, biometeorology, and mental health. The most cited article (68) employed advanced modeling techniques such as the Cause of Death Ensemble Model (CODEm) and DisMod-MR to provide estimates of mortality and life expectancy across 195 countries. It offered a benchmark for comparative health system performance analysis by establishing global trends and identifying regional disparities in mortality outcomes. Similarly (69), expanded this work by modeling the incidence and prevalence of 328 diseases and injuries. Their methodological contributions enriched the global burden of disease (GBD) modeling framework and offered critical insights for resource allocation and health service prioritization at national and global levels.

The study depicted in (70) focused specifically on cardiovascular diseases, applying GBD modeling approaches to analyze trends in the burden of these conditions across 25 years. This study provided a focused application of disease-specific modeling that supports targeted health interventions within cardiovascular care systems. Furthermore (71), contributed to health systems modeling by translating epidemiological data into Disability-Adjusted Life Years (DALYs) and Healthy Life Expectancy (HALE) metrics, which are widely used indicators in health technology assessments and health policy evaluation.

Unlike the broader focus seen in (68–71), examining the overall disease burden or environmental impacts on health, studies depicted in (72) and (73) deal with particular health issues, unmet supportive care needs in cancer patients, and sickle cell disease epidemiology, respectively, providing detailed insights into these areas. More specifically (72), contributed to modeling efforts in oncology by identifying gaps between cancer patients' supportive care needs and the services provided. Their needs-based modeling approach directly applies to health systems seeking to improve patient-centered care. The study depicted in (73) used geostatistical modeling to map the global burden of sickle cell disease in neonates, enabling targeted policy interventions and guiding service delivery planning. On the other hand (74), and (75) (papers 6 and 8) assess the impact of COVID-19. Notably, paper (75) used the Lives Saved Tool (LiST) to estimate the indirect effects of the COVID-19 pandemic on maternal and child mortality in low- and middle-income countries, illustrating its extensive ripple effects beyond the immediate health outcomes associated with the virus itself. This study exemplified system shock modeling, showing how disruptions in service delivery can be simulated to inform preparedness and resilience strategies. The study in (74) used burden-of-disease models to quantify the mental health impact of the COVID-19 pandemic, highlighting the need to incorporate psychosocial factors into public health planning. A distinctive perspective is provided in the study depicted in (76), which introduced the Universal Thermal Climate Index (UTCI). This thermal modeling tool integrates environmental data to assess human health risks, thus expanding the traditional scope of health system modeling to include climate-related stressors. The study shows an innovative approach to evaluating thermal comfort and stress, providing a unique perspective to health system modeling compared to the epidemiological or survey-based methodologies prevalent in the other studies depicted in Table 3. The study in (77) also stands out as it developed a validated self-report tool to assess experiences of discrimination, thereby facilitating the integration of social determinants into health modeling frameworks.

Regardless of their focus, all the studies in Table 3 highlight the intricate relationship between human health and various external factors such as environmental, social, or pandemic-related factors. The diversity of methodologies, from surveys and epidemiological studies to complex mathematical modeling, underscores the multifaceted nature of health research and the need for interdisciplinary approaches to address global health challenges.

#### 4.2.2 Publications with the highest number of articles

Table 4 depicts the 10 most relevant sources (journals and conference proceedings), identified by the total number of articles. These sources focused on various disciplines, including nursing, virology, HIV, psychiatry, tuberculosis and leprosy, and parasitology. As shown in the table, of the 10 most relevant sources, PLOS One was the most relevant journal with 68 articles, while the Health Policy and Planning journal was the “least” relevant with 18 articles.

**Table 4:** Top 10 most relevant sources.

Rank	Sources	Articles
1.	PLOS One	68
2.	Health Systems	45
3.	BMC Health Services Research	38
4.	BMJ Open	37
5.	BMJ Global Health	31
6.	American Journal of Health-System Pharmacy	30
7.	BMC Public Health	23
8.	The Lancet Global Health	23
9.	International Journal of Environmental Research and Public Health	22
10.	Health Policy and Planning	18

#### 4.2.3 Publications with the highest number of citations

Table 5 shows the most locally cited sources (from the reference lists).

**Table 5:** Top 10 most locally cited sources.

Rank	Source	Citations (TC)
1.	The Lancet	1,864
2.	PLOS One	1,136
3.	New England Journal of Medicine	1,014
4.	Journal of the American Medical Association	800
5.	BMJ	467
6.	PLOS Medicine	460
7.	Social Science & Medicine	447
8.	Medical Care	392
9.	Journal of Health Sciences	384
10.	Nature	375

According to (61), local citations serve as a quantitative measure that captures the frequency with which papers within a given collection reference other works in the same collection. In this study, 72,714 cited sources were included in the reference lists of the 2,023 articles. Regarding local citations, The Lancet stands out in 1st position with 1,864 citations, while in 10th position is the journal nature with 375 citations.

#### 4.2.4 Top 10 most indexed publications

Table 6 lists the most relevant sources, including the local impact of each source, the year in which each source's manuscripts were first published (PY_start), the overall number of citations (TC), the total number of publications (NP), and other measures and indices of scientific production like the h-index, the g-index, and the m-index (78).

**Table 6:** Top 10 local impact sources and most relevant sources.

Rank	Element	h_index	g_index	m_index	TC	NP	PY_start
1	PLOS One	18	35	1.125	1,334	68	2008
2	The Lancet	15	16	0.938	9,989	16	2008
3	The Lancet Global Health	15	23	1.5	2,174	23	2014
4	American Journal of Health System Pharmacy	12	21	0.444	485	30	1997
5	BMJ Global Health	11	20	1.375	426	31	2016
6	PLOS Medicine E	11	15	0.611	691	15	2006
7	BMC Public Health	10	20	0.625	426	23	2008
8	BMJ Open	10	17	0.833	329	37	2012
9	Health Systems	10	12		248	45	
10	Journal of the American Medical Informatics Association	9	14	0.563	233	14	2008

The h-index is a metric to assess individual publications' productivity and scholarly impact (78). It relates to the number of publications (N) contained within a corpus of articles, which have been ordered according to the frequency of their citation occurrences. The g-index, a derivative of the h-index, is a bibliometric measure that acknowledges scholarly articles that have garnered the highest number of citations within a specific dataset (64). The m-index is an alternative to the h-index and represents the h-index for each year starting from the initial publication (79).

The results indicate that PLOS One has the highest h-index of 18, followed by The Lancet and The Lancet Global Health, with an h-index of 15. Although The Lancet Global Health has a higher g-index of 23 than The Lancet with 16, the latter is still highly rated because the h-index tends to consider both the highest number of publications and the highest number of citations, lowering the index compared to the g-index and m-index (79).

### 4.3 Most productive and influential countries and authors in health systems modeling

**Figure 2:**
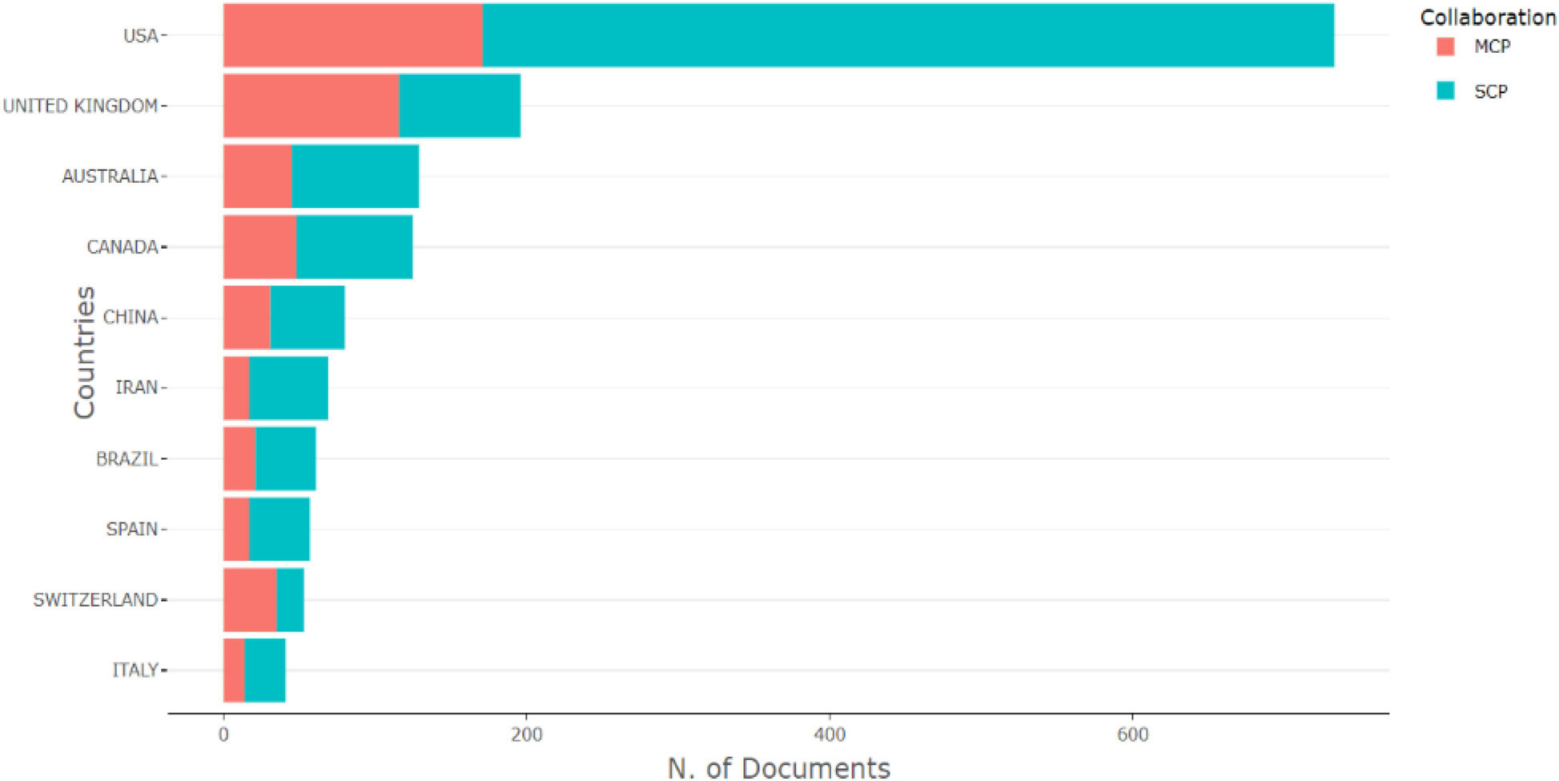
Data visualization of countries of origin of corresponding authors.

The countries of origin of corresponding authors in health systems modeling research are presented in Table 7 and Figure 2. A corresponding author is the primary contact with the journal's editor and represents all co-authors. Collaboration patterns are captured through two indices: Single-Country Publications (SCP), which reflect intra-country collaboration, and Multiple-Country Publications (MCP), which indicate inter-country collaboration. The MCP counts articles co-authored by individuals from institutions in different countries, while the SCP includes publications with co-authors from the same country.

**Table 7:** Countries of origin of corresponding authors.

Country	Articles	SCP	MCP	Freq	MCP_Ratio
USA	733	562	171	0.362	0.233
UK	196	80	116	0.097	0.592
Australia	129	84	45	0.064	0.349
Canada	125	77	48	0.062	0.384
China	80	49	31	0.040	0.388
Iran	69	52	17	0.034	0.246
Brazil	61	40	21	0.030	0.344
Spain	57	40	17	0.028	0.298
Switzerland	53	18	35	0.026	0.660
Italy	41	27	14	0.020	0.341

Table 7 and Figure 2 highlight the top 10 contributing countries (out of 90) to health systems modeling research between 1992 and 2023, based on total citations and SCP/MCP outputs. The United States (733 corresponding authors), the United Kingdom (196), Australia (129), Canada (125), and China (80) lead in terms of combined intra- and inter-country collaboration. A clear correlation exists between high SCP and high MCP scores, suggesting that countries with strong domestic research capacity are well-positioned in international collaborations. Citation frequency follows a similar pattern, reinforcing the influence of these leading nations, with the USA consistently occupying the top slot. Notably, the UK displays a higher MCP than SCP, indicating a strong international orientation in its research collaborations.

The bibliometric analysis identifies the USA and UK as gatekeepers within the global health systems modeling network. The USA, with 562 SCP and 171 MCP publications, and the UK, with an MCP ratio of 0.592, are the most important connectors influencing research agendas, funding, and publication dynamics. Their central roles in the collaboration network emphasize their strategic positioning as global research hubs. Conversely, countries such as Iran (MCP ratio = 0.246), Spain (0.298), and Italy (0.341) are relatively under-connected in this network. Despite their contributions, these lower MCP ratios suggest limited international collaboration, which may stem from barriers such as restricted access to international funding, institutional limitations, language differences, or fewer established global partnerships.

On the other hand, countries like Switzerland (MCP ratio = 0.660), Brazil (0.344), and China (0.388) represent emerging research communities with growing international engagement. Switzerland, in particular, stands out for its high MCP ratio despite a modest publication count (53 articles), signaling its increasing integration and influence within global research networks. Brazil and China, with rising publication outputs and moderate international collaboration, also show strong potential to expand their global research presence further.

Table 8 lists the top 10 authors based on the h-index. Blakely T. and Hay S.I. are the top authors in the field of health systems modeling for the period 1992–2023. Blakey T. is in the top position because of a higher g-index and the number of publications. The author also started to publish in 2009, as opposed to Hay S.I., who was in the second position, which started earlier in 2006. Although Wilson N. has the second most publications (NP = 25), he is in the fourth position because of a lower h-index than Blakely (15), Hay (15), and Kvizhnadze (12). It has been suggested that the h-index predicts future scientific achievements more accurately than other research metrics (79).

**Table 8:** Author's local impact.

Rank	Author	h_index	g_index	m_index	TC	NP	PY_start
1	Blakely T	15	24	1	609	26	2009
2	Hay S.I.	15	18	0.833	11,511	18	2006
3	Kvizhnadze G.	12	16	1.2	380	16	2014
4	Wilson N.	12	21	1.333	459	25	2015
5	Barnighausen T.	11	16	0.917	9,714	16	2012
6	Gupta R	11	12	1.375	17,599	12	2016
7	Gething P.W.	10	12	0.556	8,350	12	2006
8	Cleghorn C.L.	9	11	1	300	11	2015
9	Mokdad A.H.	9	10	0.818	8,172	10	2013
10	Naghavi M.	9	10	0.818	10,060	10	2013

TC, total citations; NP, number of publications; PY_start, publication year start.

### 4.4 Most productive and influential affiliations in health systems modeling

A total of 4,069 institutions contributed to health systems modeling research over the 32 years. The 10 most productive institutions are presented in Table 9, ordered according to the number of publications by each institution. The findings are compared to the Academic Ranking of World Universities (ARWU) 2022 (80) and QS World University Rankings 2023 (81), which are internationally recognised rankings of universities in terms of reputation and excellence. Surprisingly, the Tehran University of Medical Sciences in Iran has the highest number of publications (132), although its ARWU ranking is between 401 and 500. The University of Washington in the USA is second with 128 articles, followed by the London School of Hygiene & Tropical Medicine in the UK with 123 articles. Except for one institution in Canada (ranked 9th with 91 articles), all the remaining seven were either in the USA or the UK.

**Table 9:** Most relevant affiliations.

Rank	Affiliation	Country	Articles	ARWU 2022	QS 2023
1	Tehran University of Medical Sciences	Iran	132	401–500	-
2	University of Washington	USA	128	17	80
3	London School of Hygiene & Tropical Medicine	UK	123	151–200	-
4	University of Pittsburgh	USA	119	82	181
5	Duke University	USA	115	31	50
6	Harvard University	USA	110	1	5
7	University of Oxford	UK	108	7	4
8	Imperial College London	UK	95	23	6
9	University of Toronto	Canada	91	22	34
10	Johns Hopkins University	USA	85	14	24

All the institutions and research centres in Table 9 are ranked at least in one of the two world university rankings (ARWU or QS). While the Tehran University of Medical Sciences is perceived to be in the 401–500 position on ARWU 2022 rankings, it has published many articles on health systems modeling. The ARWU's number one institution (Harvard University), number 5 on the QS ranking, has fewer publications (110) in health systems modeling. As noted, the dominance of institutions from the USA, followed by those from the UK, is apparent as these two countries have eight out of the 10 institutions within the top 10 slots. The noticeable inclusion of Iran and Canada in health systems modeling is a positive development.

### 4.5 Most productive and influential countries in health systems modeling

Table 10 reflects the countries that produced the top 10 highest number of citations within the period under review. In the corpus of selected articles, many authors cited scientific articles originating from the USA, which amassed 24,467 citations. The UK emerged as the second most frequently cited country with 5,139 citations. Australia also received a notable share of citations, with its scientific output accruing 3,361 citations and exhibiting an average article citation of 26.054. Remarkably, South Africa was featured among the top 10 most frequently cited countries, with 744 articles on the subject matter.

**Table 10:** Most cited countries.

Rank	Country	Total Citations (TC)	Average Article Citations
1	USA	24,467	33.379
2	UK	5,139	26.219
3	Australia	3,361	26.054
4	Canada	2,340	18.720
5	Germany	1,181	39.367
6	Switzerland	1,089	20.547
7	South Africa	744	18.600
8	Iran	704	10.203
9	Brazil	670	10.984
10	China	657	8.213

### 4.6 Three-field plot: countries, keywords, and sources

**Figure 3:**
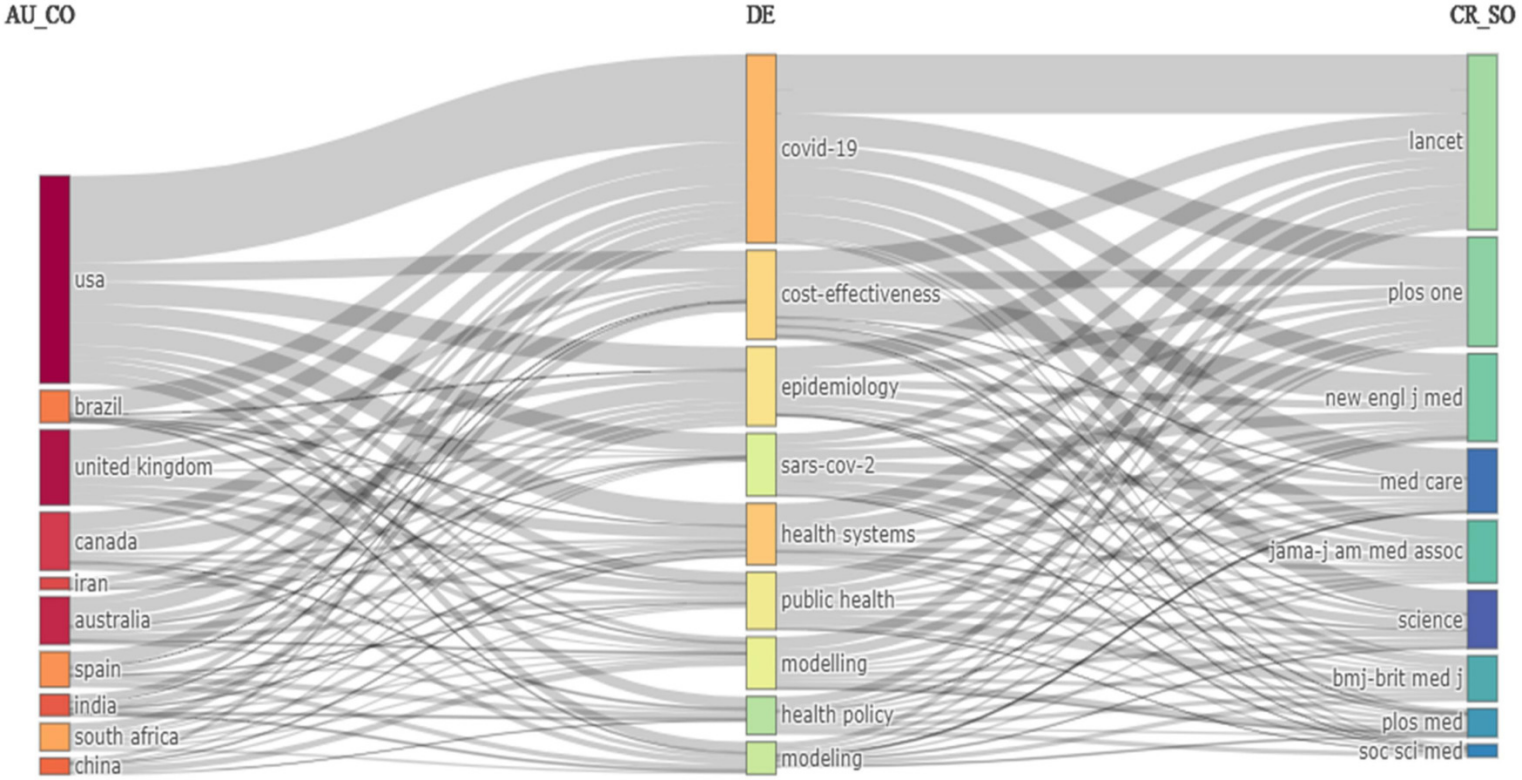
Data visualization of the three-field plot.

The Sankey Plot depicted in Figure 3 illustrates the relationship between the top countries (left field), top keywords (middle field), and top sources (right field). Based on a Sankey diagram, a three-field plot can be used to interpret the proportion of highly used keywords to top countries that use them and the top publishers within the subject. The flows and related quantities are illustrated in proportion to one another on the Sankey diagram. The magnitudes are represented by the width of the lines or arrows; the wider the line, the stronger the flow. The flow symbols can be merged or divided at every process phase along their pathways. Colour can be used to categorise the diagram or to indicate the change from one state of the process to another.

Specifically, the diagram visually depicts how each of the top ten countries connects to the primary keywords they have employed in their publications and the journals in which these articles appeared. Wider lines between countries, keywords, and sources indicate greater frequency or stronger associations. For instance, the USA and UK exhibit robust flows toward the keyword “COVID-19,” signifying extensive research output in this area, primarily disseminated through leading journals such as The Lancet and PLOS One. Conversely, narrower lines highlight more modest associations, such as those observed for countries like South Africa, indicating either lower volume or narrower topical focus in their contributions within these top journals.

A closer look at low- and middle-income countries (LMICs) in the plot, namely Brazil, Iran, India, South Africa, and China, reveals meaningful but less dominant contributions to health systems modeling research. These countries exhibit active engagement with key thematic areas such as “COVID-19,” “public health,” and “modeling,” suggesting that LMICs are contributing to globally relevant and urgent topics. For instance, Brazil and India show visible connections to “cost-effectiveness” and “public health,” which may reflect their ongoing efforts to optimize limited healthcare resources. Iran and South Africa are linked to narrower keyword ranges, while China shows more diverse connections to terms like “health systems” and “epidemiology.” Regarding publication venues, LMICs tend to publish in widely recognized outlets such as PLOS One, BMJ, although their link strength is lower than in high-income countries. These findings emphasize the importance of supporting broader inclusion of LMICs in international health systems modeling research, particularly given their unique perspectives and contextual challenges.

Except for South Africa, all 10 countries (the USA, Brazil, the UK, Canada, Iran, Australia, Spain, India, and China) made noticeable contributions related to COVID-19. The subject was also published in all the top journals in terms of citations identified in Table 5 (The Lancet, PLOS One, New England Journal of Medicine, Medical Care, Journal of the American Medical Association, Journal of Health Sciences, BMJ, PLOS Medicine, Nature, and Social Science & Medicine). The Lancet stands out as the most published journal, covering all the nine most used authors' keywords (DE) identified: COVID-19, cost-effectiveness, epidemiology, SARS-CoV-2 (coronavirus), health systems, public health, modeling, and health policy.

### 4.7 Most frequently used keywords

An important approach for determining trending areas and scholarly focus is frequently used keywords (54). The bibliometric analysis also included an analysis of the frequently used keywords. Using another bibliometric tool, VOSviewer, the keywords were ranked according to their total link strength (TLS) and the number of times they occurred. The TLS indicator reflects the strength of all collaborations between a specified researcher and other researchers (82). Table 11 reflects the most frequently used keywords in the research, ranked according to the TLS. As displayed in the table, the keyword “care” has the highest TLS (1,060) and the highest number of occurrences (213). This is followed by “impact” with a TLS of 789 and 161 occurrences. The keyword “health care” has the lowest TLS (474) but the third highest number of occurrences (144). All the keywords are relevant within the health research area. The occurrence of keywords such as “care,” “health,” and “health-care,” individually and in combination, is attributed to the inherent scope and nature of health systems modeling literature. Researchers frequently employ these terms both broadly and specifically; “health” often denotes overarching population outcomes and conditions, “care” emphasizes direct service delivery or interventions, while “health-care” commonly represents the healthcare sector holistically. This lexical variety reflects different research emphases and highlights the interdisciplinary and multifaceted nature of the health systems modeling field, aligning closely with the thematic breadth identified in the bibliometric analysis.

**Table 11:** Most frequently used keywords.

Rank	Keyword	Occurrences	Total Link Strength (TLS)
1	Care	213	1,060
2	Impact	161	789
3	Mortality	130	695
4	Risk	119	589
5	Health	130	588
6	Cost-effectiveness	100	571
7	Outcomes	92	507
8	Prevention	81	500
9	Management	101	485
10	Health-care	144	474

**Figure 4:**
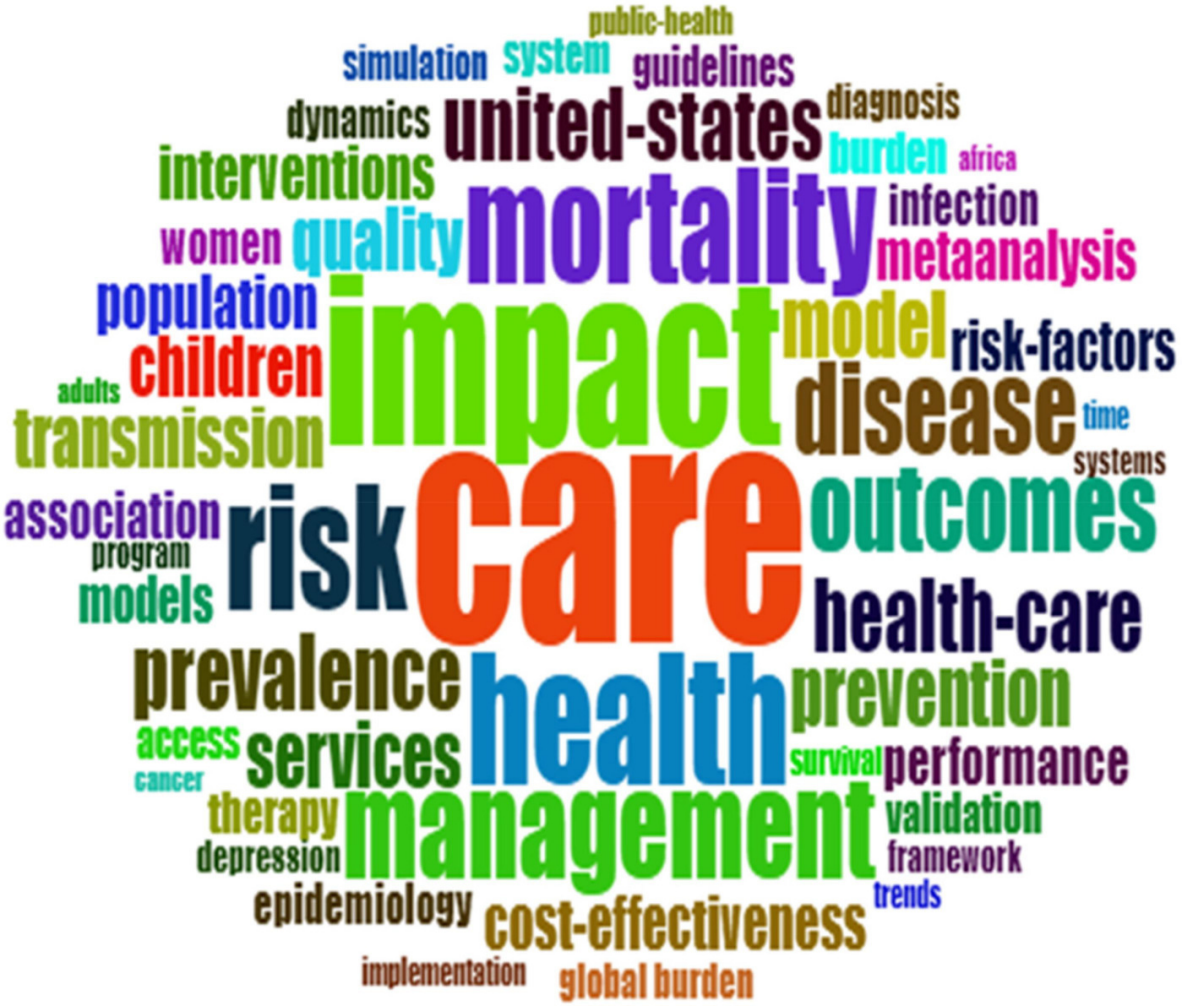
Word cloud showing the keywords used.

**Figure 5:**
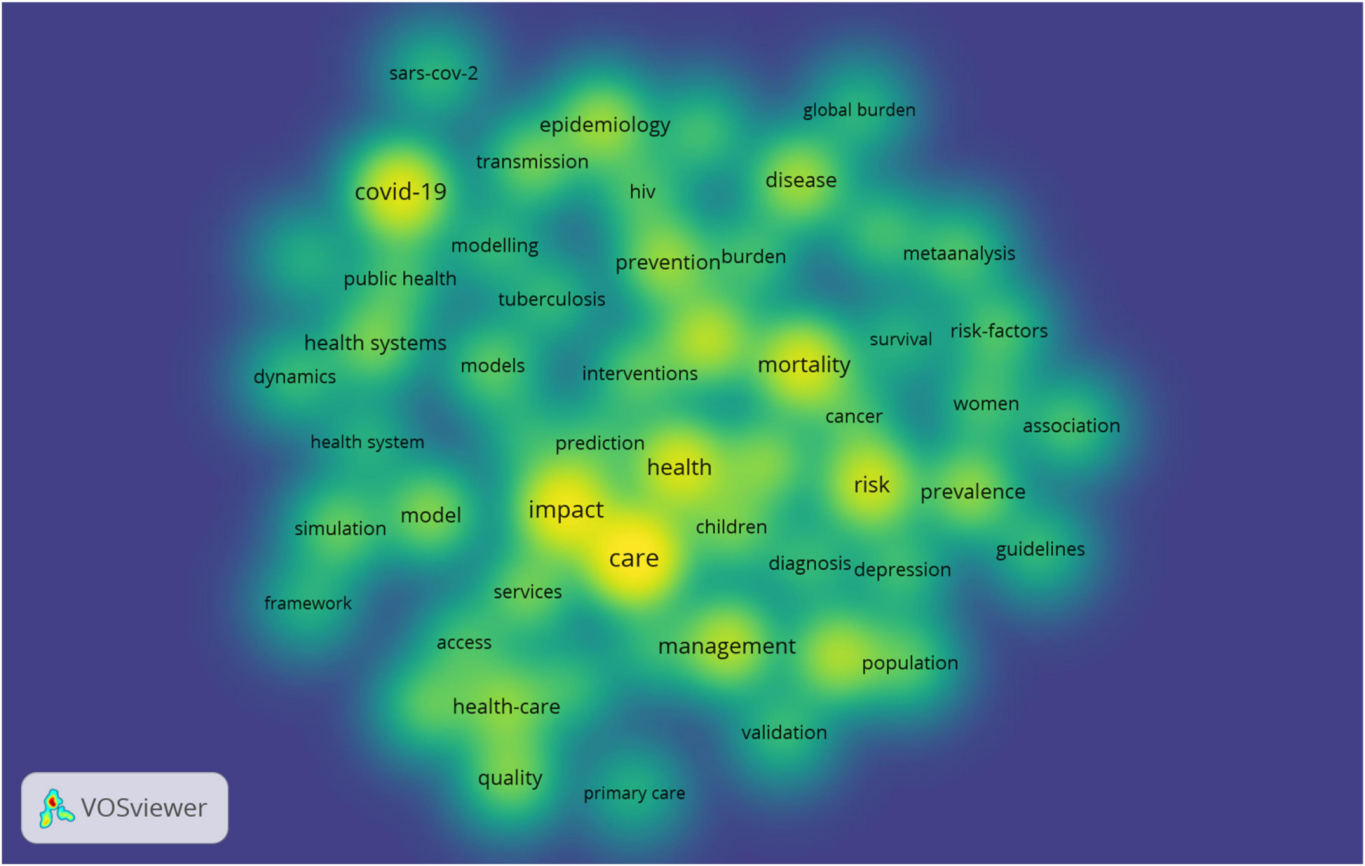
Co-occurrence of the keywords (overlay density visualization).

Word clouds can also be used to graphically illustrate frequently used keywords. A word cloud refers to a graphic that depicts word frequency as a quick method to determine the main idea of written content (14). The keywords that appear larger in the graphic represent the most frequently used keywords in the analysed text. The analysis of the content of the 2,023 documents produced 4,163 keywords, which encompassed, among others, terminologies within the healthcare industry, the countries involved, the various techniques used, the participants within the various articles, and the inputs and outputs from the articles. These are depicted in Figures 4, 5. The figures graphically depict the most frequently occurring keywords such as those appearing in Table 11 as well as others used in health systems modeling including “healthcare”, “disease”, “prevalence”, “model”, “transmission”, “interventions”, “health systems”, “population”, “transmission” and “quality”.

### 4.8 Thematic map of the field

**Figure 6:**
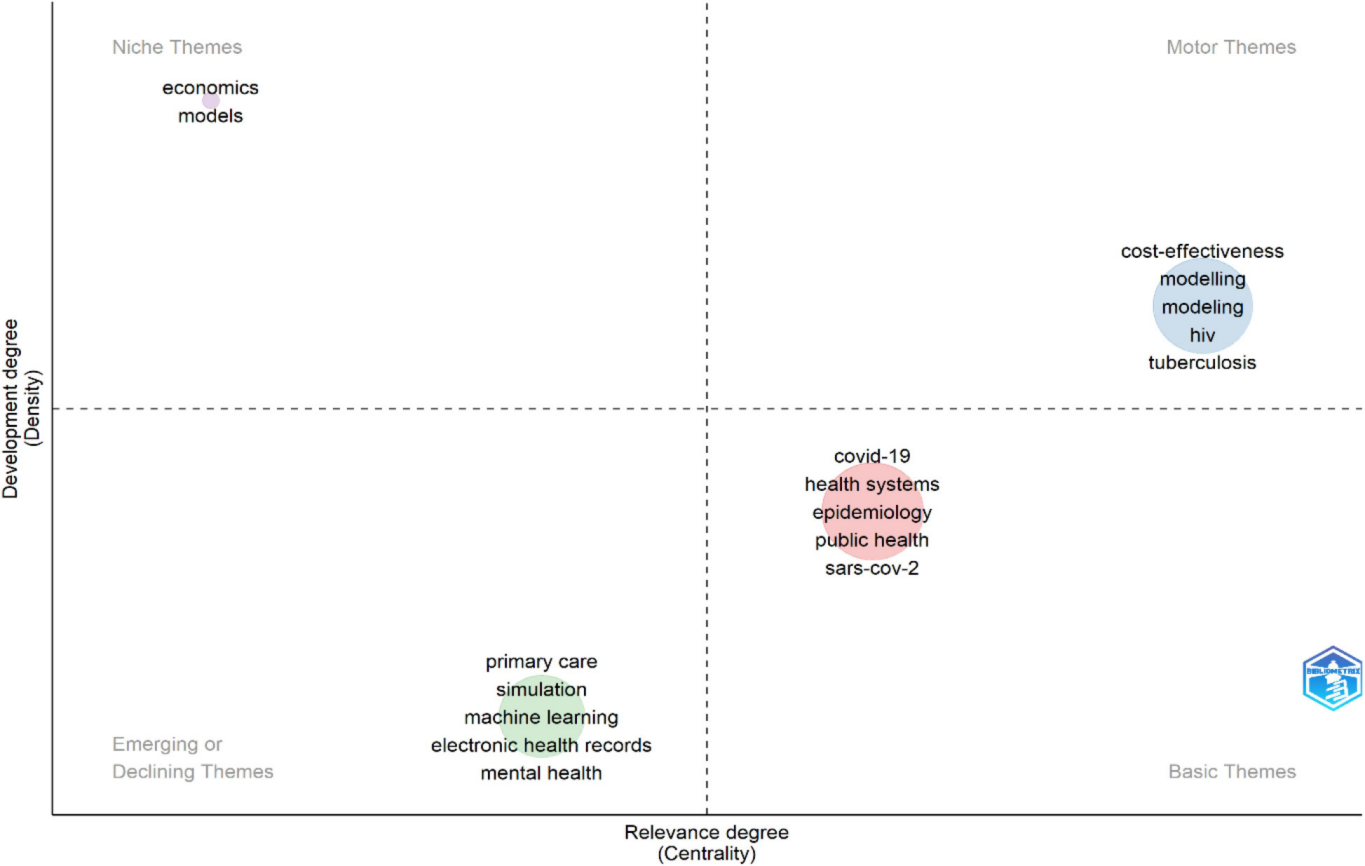
Thematic map.

A thematic map is used to generate insight into the status quo of current research in a particular field and the prospects of sustaining future research (54). Thematic analysis is helpful in enlightening scholars and stakeholders about the possibility of creating novel thematic research topics within a particular discipline (52). A thematic map illustrates how the study themes have changed over time in bibliometric analysis. It is based on bibliometric data that details the frequency and co-occurrence of keywords, authors, journals, and other bibliographic variables in scientific publications (52). Each point on a thematic map represents a research topic, and the interval between the points indicates how related the topics are to one another (54). The similarities between topics increase as they become more contiguous. The size of the points reflects how frequently the topic appears in the literature. The main research themes in a discipline may be found, and their history can be understood by looking at a thematic map. Additionally, one can spot developing topics and follow their evolution over time (54). This data can be employed to direct research efforts, pinpoint research gaps, and drive policy choices. A thematic map with four quadrants is depicted in Figure 6.

Quadrant 1 (the upper right quadrant) represents the motor themes, which are highly developed and mature (driving themes) (55). Quadrant 1 reflects “cost-effectiveness,” “modeling,” “modeling,” “HIV,” and “tuberculosis.” Quadrant 2 (the bottom right quadrant) resembles the basic themes that are generally growing but have not reached maturity, as in Quadrant 1 (54). This quadrant contains themes with high density but low centrality. These themes might be growing, but still require more investment to mature. The themes “economics” and “models” are growing themes that still demand considerable investment to mature. They focus on economic modeling techniques within health systems. In Quadrant 3 (the top left quadrant), the niche themes are more developed, although they are not yet well connected to other themes within the field (64). They have low centrality and density, suggesting they are either at an early exploration stage or losing momentum. “Primary care,” “simulation,” “machine learning,” “electronic health records,” and “mental health” are depicted as the developed themes that were yet to be well-correlated to other themes within the field of systems health modeling. Emerging trends like machine learning and electronic health records show the potential to grow as they address modern healthcare needs. Quadrant 4 (the bottom left quadrant) represents themes that are either emerging or declining (disappearing) (61). Basic themes exhibit high centrality but low density, making them foundational yet not deeply explored. They underpin the field and show promise for future research. “COVID-19,” “health systems,” “epidemiology,” “public health,” and “SARS-CoV-2” have either declined in terms of scientific research or scholars are now gaining interest in them.

Table 12 enumerates the terms for each cluster according to their frequency. The table used author keywords with the Leiden clustering algorithm to identify research clusters. The analysis used a minimum cluster frequency of 5 per thousand documents, with a complete counting method and a minimum of 5 labels per cluster. This approach revealed six distinct clusters comprising 63 items, interconnected through 403 links with a cumulative total link strength of 727. This comprehensive clustering approach emphasises the complex relationships and thematic congruence within health systems modeling.

**Table 12:** Cluster keywords.

Cluster 1	Cluster 2	Cluster 3
Breast cancer	Cancer	Computational modeling
Cost-effectiveness	Diabetes	Coronavirus
Cost-effectiveness analysis	Electronic health record	Depression
Economic evaluation	Forecasting	Discrete event simulation
HIV	Iran	e-health
Implementation	Machine learning	Health system
Malaria	Modeling	Healthcare
Modeling	Models	Mental health
Policy	Mortality	Simulation
Prevention	Prediction	Structural equation modeling
Quality of life	Predictive modeling	System dynamics
Screening	Primary care	Telemedicine
South Africa	Primary health care	
Stroke	Quality improvement	
Treatment	Risk factors	
Tuberculosis		
Cluster 4	Cluster 5	Cluster 6
Epidemiology	Cost	Brazil
Global health	Decision making	Covid-19
Health economics	Economics	Mathematical modeling
Health policy	Health services	Sars-cov-2
Health services research	Hospitals	Vaccination
Health systems	India	
Mathematical modeling	Pandemic	
Public health		

Table 12 reveals the following clusters and overlapping clusters:

**Cluster 1:** Focuses on public health challenges and their solutions, including breast cancer, tuberculosis, malaria, and HIV. It also emphasises cost-effectiveness analysis, screening, and improving quality of life through prevention, treatment, and healthcare policy, particularly in regions like South Africa.
**Cluster 2:** Centers on healthcare technology and predictive analytics. Topics include machine learning, predictive modeling, and electronic health records. It also covers healthcare issues like diabetes, cancer, and risk factors, emphasizing improving primary care and quality improvement.
**Cluster 3:** Explores healthcare systems and simulation-based modeling techniques. Key topics include discrete event simulation, system dynamics, and telemedicine. It also addresses mental health, coronavirus, and advances in structural equation modeling for health system improvement.
**Cluster 4:** Focuses on global health and health system research. Topics include epidemiology, public health, health policy, and mathematical modeling to analyze and improve health outcomes globally. It highlights research in health economics and health systems.
**Cluster 5:** Centers around economic factors in healthcare, including decision-making, costs, and the impact of health services on broader issues like pandemics and hospitals. It also touches on economics and regional considerations, such as India's.
**Cluster 6:** Deals with COVID-19 and related modeling efforts. Topics include SARS-CoV-2, vaccination, and the role of mathematical modeling in understanding and controlling the pandemic. It also highlights Brazil as a specific region of focus.

#### 4.8.1 Overlapping clusters

The following overlaps were also noted on the clusters:

1. Common themes across clusters: Several clusters share similar keywords and focus areas, indicating overlaps in research or application domains:
  • Health systems and policy: Clusters 1, 4, and 5 focus on health policy, health economics, and public health, emphasizing the importance of structured systems for better health outcomes. For instance, Cluster 4 includes “health systems” and “health economics,” while Cluster 1 highlights “policy” and “quality of life.” Cluster 2 also touches on healthcare systems through “primary care” and “quality improvement.”
  • Modeling and simulation: Clusters 3, 4, and 6 focus on modeling approaches such as “mathematical modeling,” “simulation,” and “structural equation modeling.” This overlap suggests a shared interest in using computational models for health decision-making and predictive analysis.
  • Economic evaluation: Clusters 1, 4, and 5 overlap in exploring the economic implications of healthcare through topics like “cost-effectiveness analysis,” “economic evaluation,” and “decision making.” This highlights a shared emphasis on optimizing healthcare investments.
2. Disease-specific overlaps: Some clusters address similar diseases or health challenges:
  • Chronic and infectious diseases: Cluster 1 focuses on diseases like HIV, tuberculosis, and stroke, while Cluster 2 addresses diabetes and cancer. These clusters emphasize treatment and prevention from slightly different perspectives (economic and primary care in Cluster 2 vs. broader policy and implementation in Cluster 1).
  • COVID-19 and related topics: Clusters 3 and 6 overlap on topics like “coronavirus,” “COVID-19,” and “vaccination.” Cluster 3 emphasizes healthcare systems and telemedicine, while Cluster 6 focuses on pandemic modeling and vaccine-related issues.
3. Technology and data in healthcare: Clusters 2, 3, and 6 share an interest in leveraging technology and data for healthcare improvements:
  • Predictive analytics and machine learning: Cluster 2 highlights “machine learning,” “predictive modeling,” and “electronic health records,” while Cluster 3 includes “telemedicine” and “system dynamics.” These technologies support decision-making and improve healthcare delivery.
  • Simulation and modeling techniques: Cluster 3's focus on “discrete event simulation” overlaps with Cluster 6's emphasis on “mathematical modeling” for pandemic responses, illustrating the role of computational methods in health crises.
4. Geographic and regional focus: Clusters 1, 5, and 6 emphasise regional health challenges:
  • Cluster 1 highlights “South Africa” as a key region, while Cluster 5 includes “India” and “pandemic,” and Cluster 6 mentions “Brazil.” These clusters overlap in addressing how regional contexts impact healthcare policies, modeling, and interventions.

These overlaps suggest a multidisciplinary and global approach to addressing health challenges, highlighting the need for integrated research and policy frameworks.

### 4.9 Additional intellectual structures

To further identify and elucidate the intellectual structures, this section discusses the co-authorship, co-citation, and bibliographic coupling networks conducted using VOSviewer.

**Figure 7:**
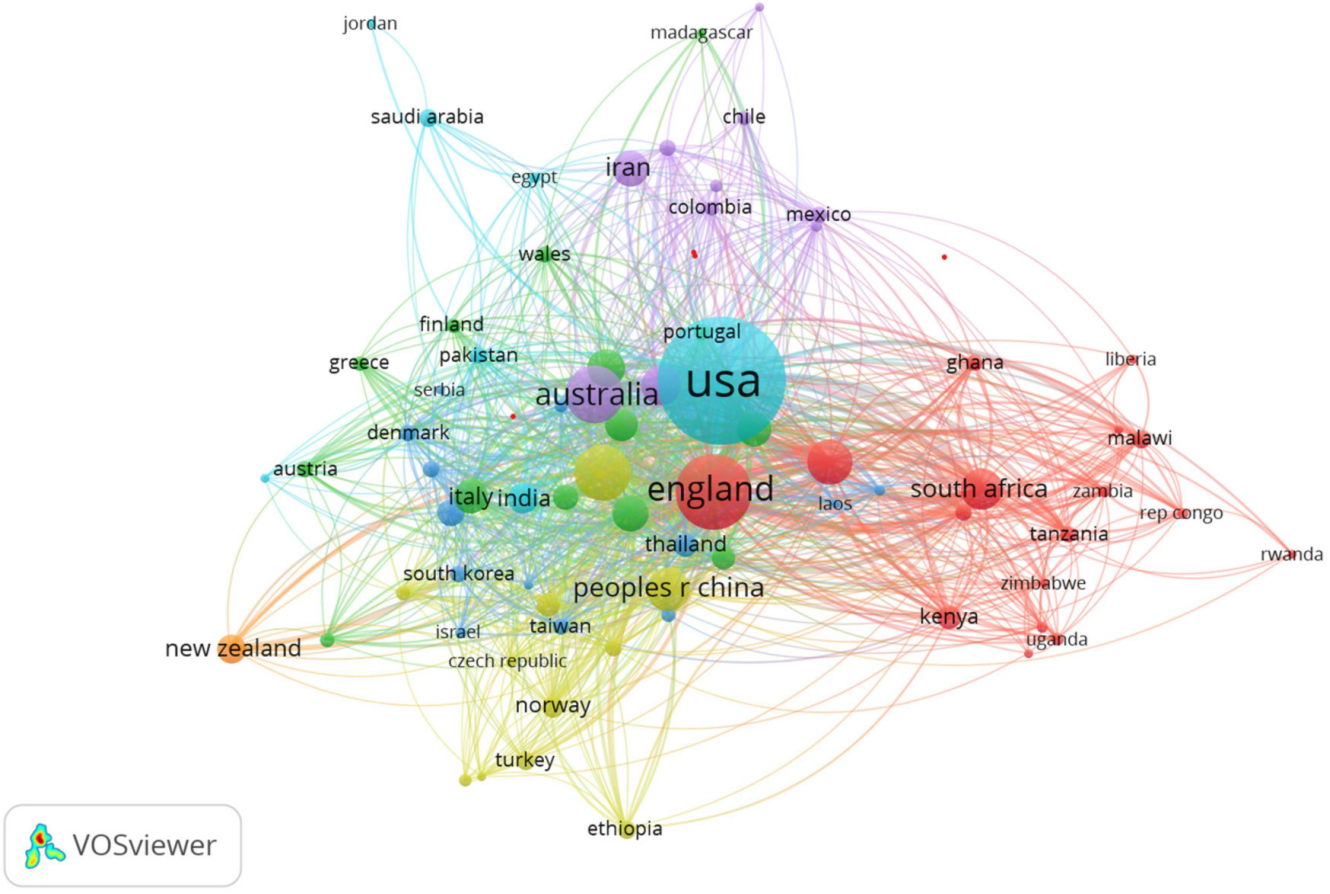
Country co-authorship.

**Figure 8:**
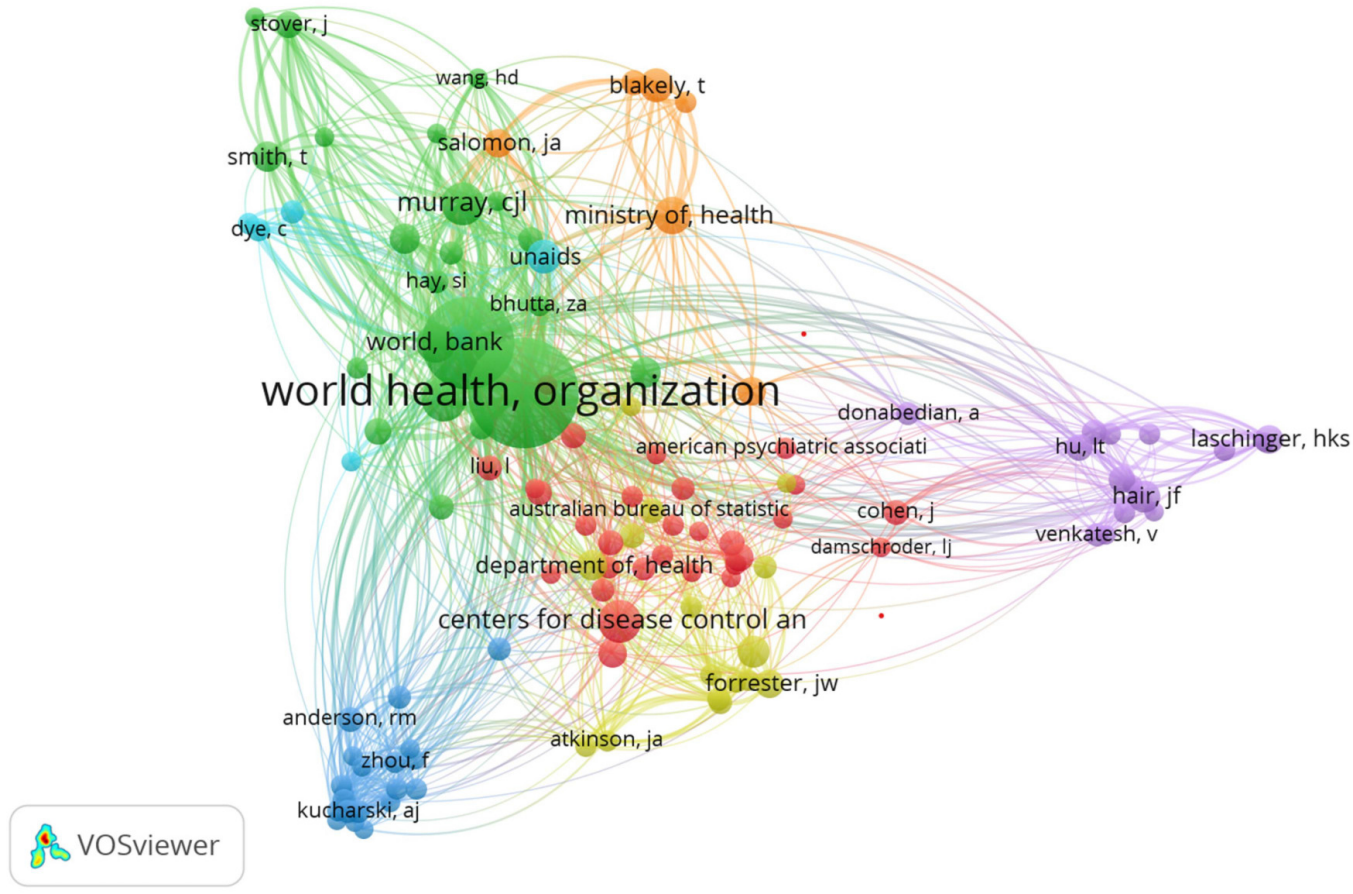
Institutional co-authorship.

Figure 7 visualization reveals the international collaboration landscape in health systems modeling. Unsurprisingly, the United States emerges as a dominant node with extensive global linkages, followed by the United Kingdom and Australia. The thickness of connecting lines indicates strong bilateral partnerships, while the presence of clusters reveals regionally cohesive research communities. Equally, to depict the interconnectedness among institutions involved in health systems modeling, Figure 8 showcases the institutional alliances. Key academic and research hubs such as the University of Washington and Tehran University of Medical Sciences are prominently positioned, reflecting prolific output and collaboration. While institutional partnerships within countries are robust, the figure highlights significant cross-border affiliations. Importantly, this co-authorship network accentuates how institutional alliances fuel interdisciplinary research, with the structure indicating both central leadership and peripheral but growing contributors.

**Figure 9:**
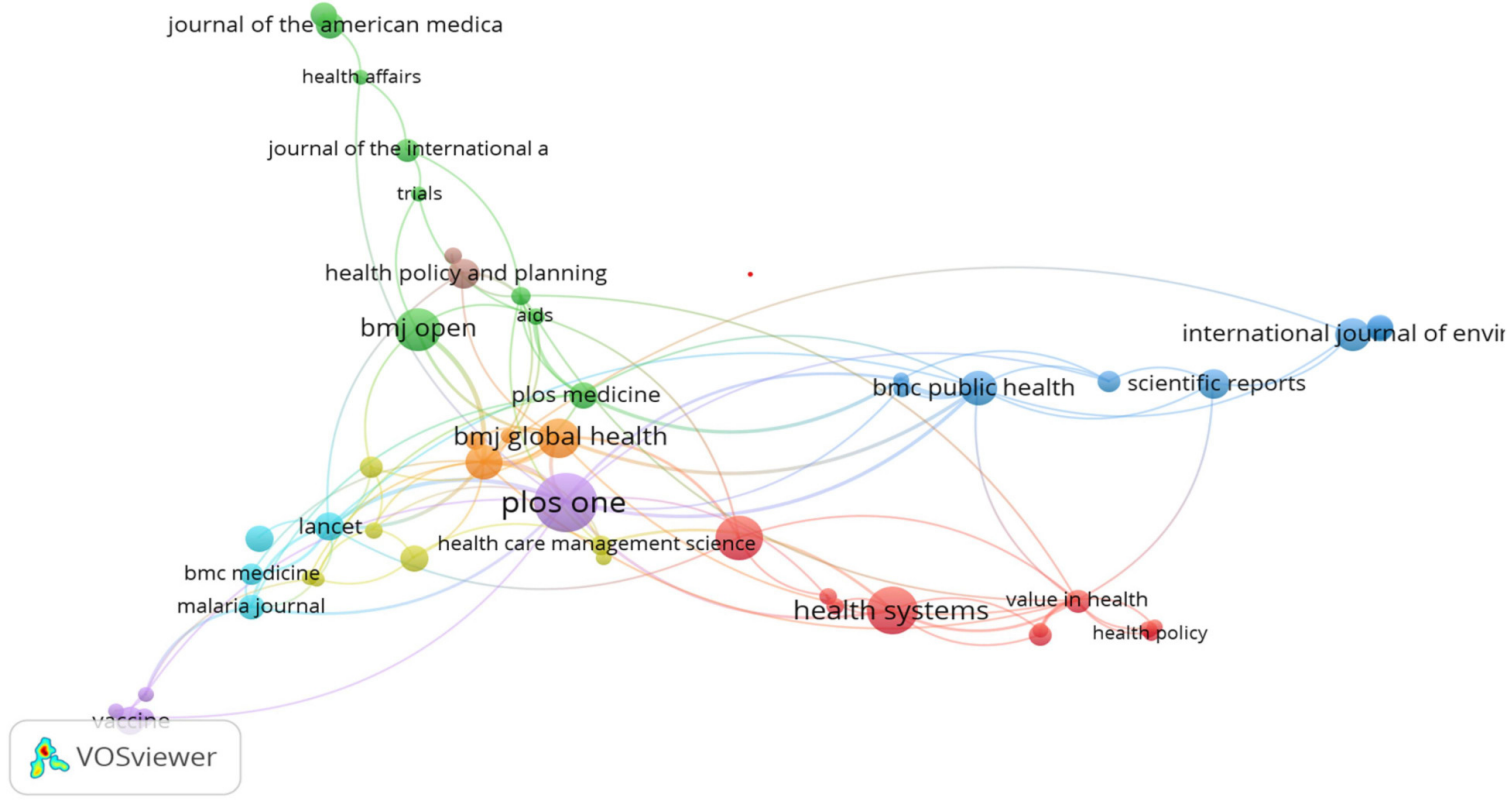
Sources co-citation.

The study also mapped the intellectual influence of journals based on how frequently they are cited together, as shown in Figure 9. Leading journals like The Lancet and PLOS One occupy central positions, indicating foundational status. The dense saturation observed suggests high cohesion around certain core outlets. These visualizations highlight epistemic dependencies and shared knowledge bases in health systems research.

**Figure 10:**
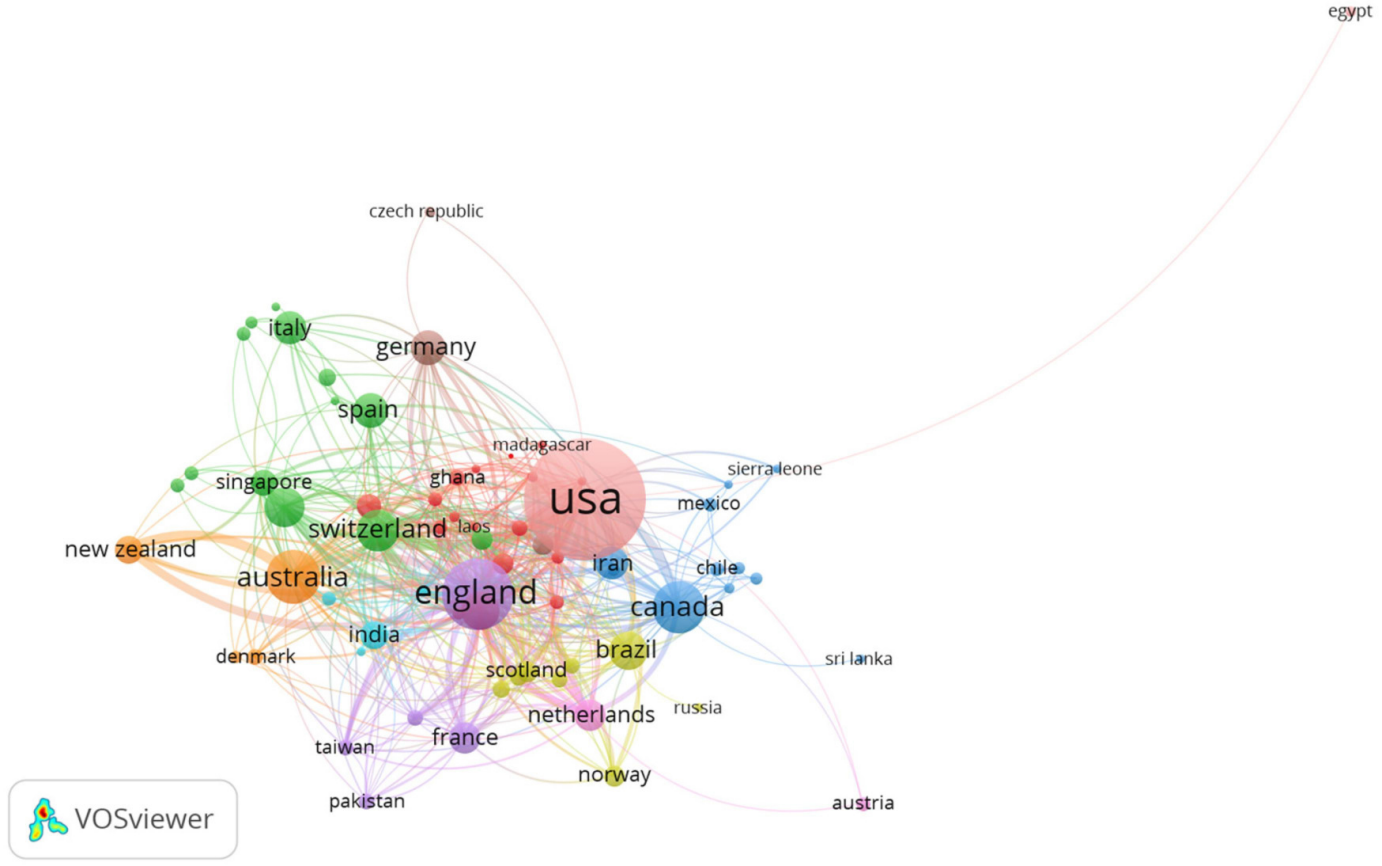
Country co-citation.

Country-level co-citation patterns are effectively highlighted in Figure 10, revealing how national research outputs are interlinked through shared citations. The USA, UK, and Australia form the triadic core, indicating their significant academic influence. The map also reveals emerging scholarly alignment among countries such as Iran and China, reinforcing global diversification. While centrality demonstrates intellectual leadership, peripheral countries, though less connected, show promising citation visibility.

**Figure 11:**
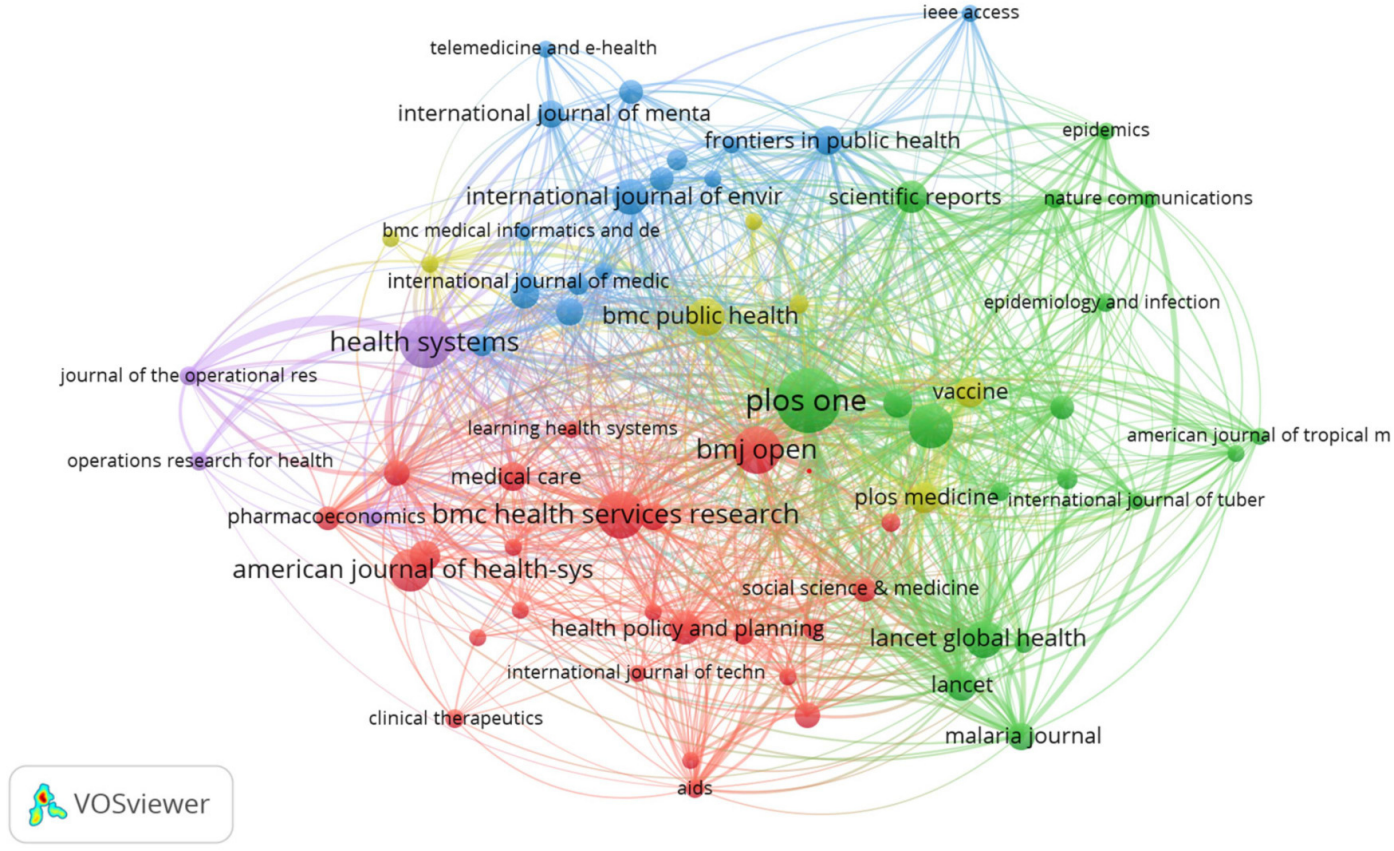
Source bibliographic coupling network.

The bibliographic coupling analysis in Figure 11 identifies journals that cite similar sources, implying thematic or methodological alignment. Notable journals like BMJ, PLOS One, and BMC Health Services Research are tightly linked, demonstrating a shared intellectual foundation. This coupling provides insight into the publication ecosystem and can guide authors seeking suitable publication venues. Additionally, the clustering of sources reveals disciplinary convergence zones and may indicate emerging niches within health systems modeling literature.

**Figure 12:**
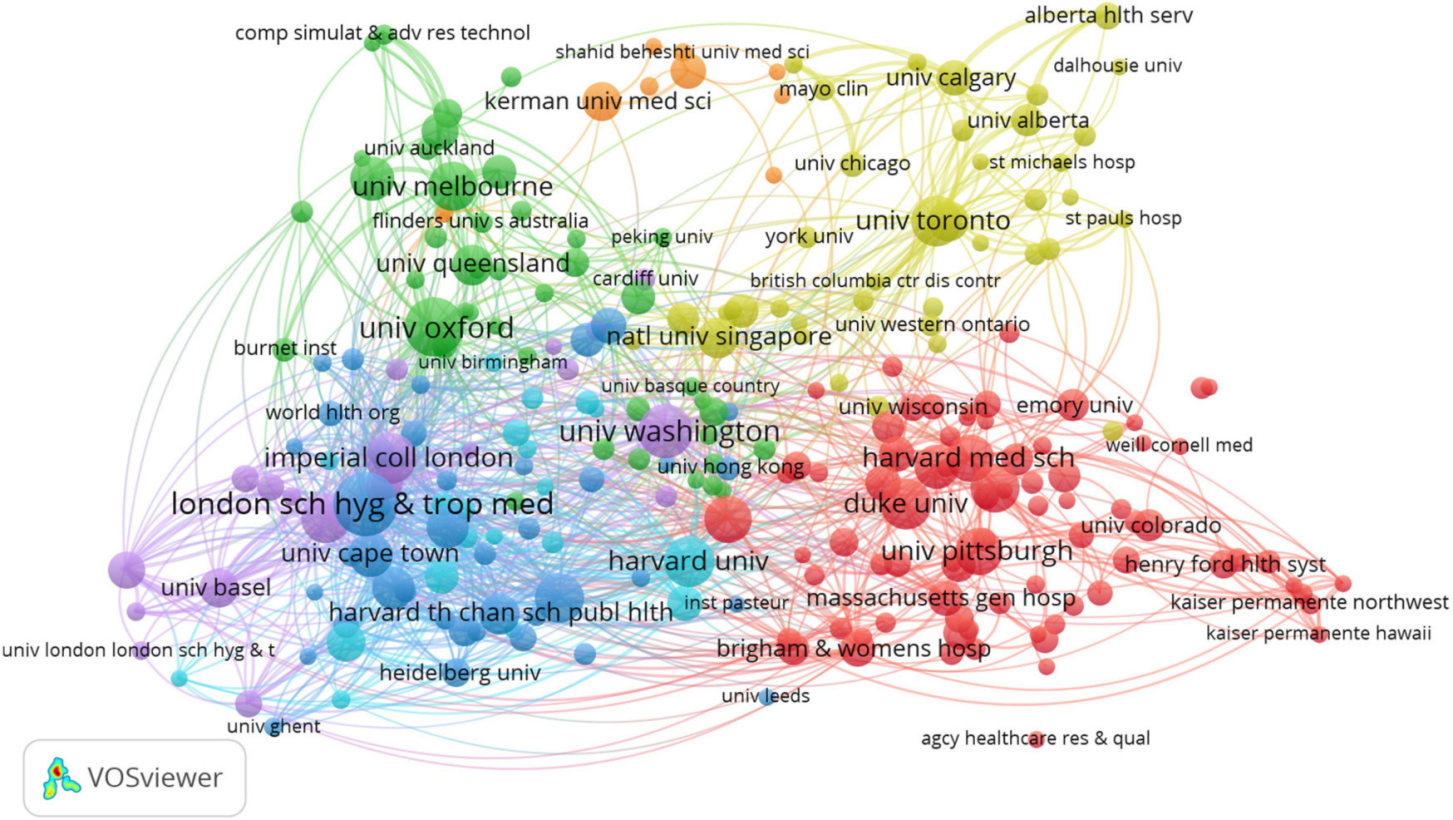
Institutional bibliographic coupling network.

Figure 12 maps institutions that exhibit similar citation behaviors, signaling aligned research interests. Institutions like Harvard, Oxford, and Tehran University of Medical Sciences form tight bibliographic clusters, reflecting thematic or methodological synergy. The network shows which institutions are thematically aligned and where collaborative potential may exist. Thus, this figure reflects research alignment and is a strategic tool for identifying potential institutional partnerships for future collaborative studies.

**Figure 13:**
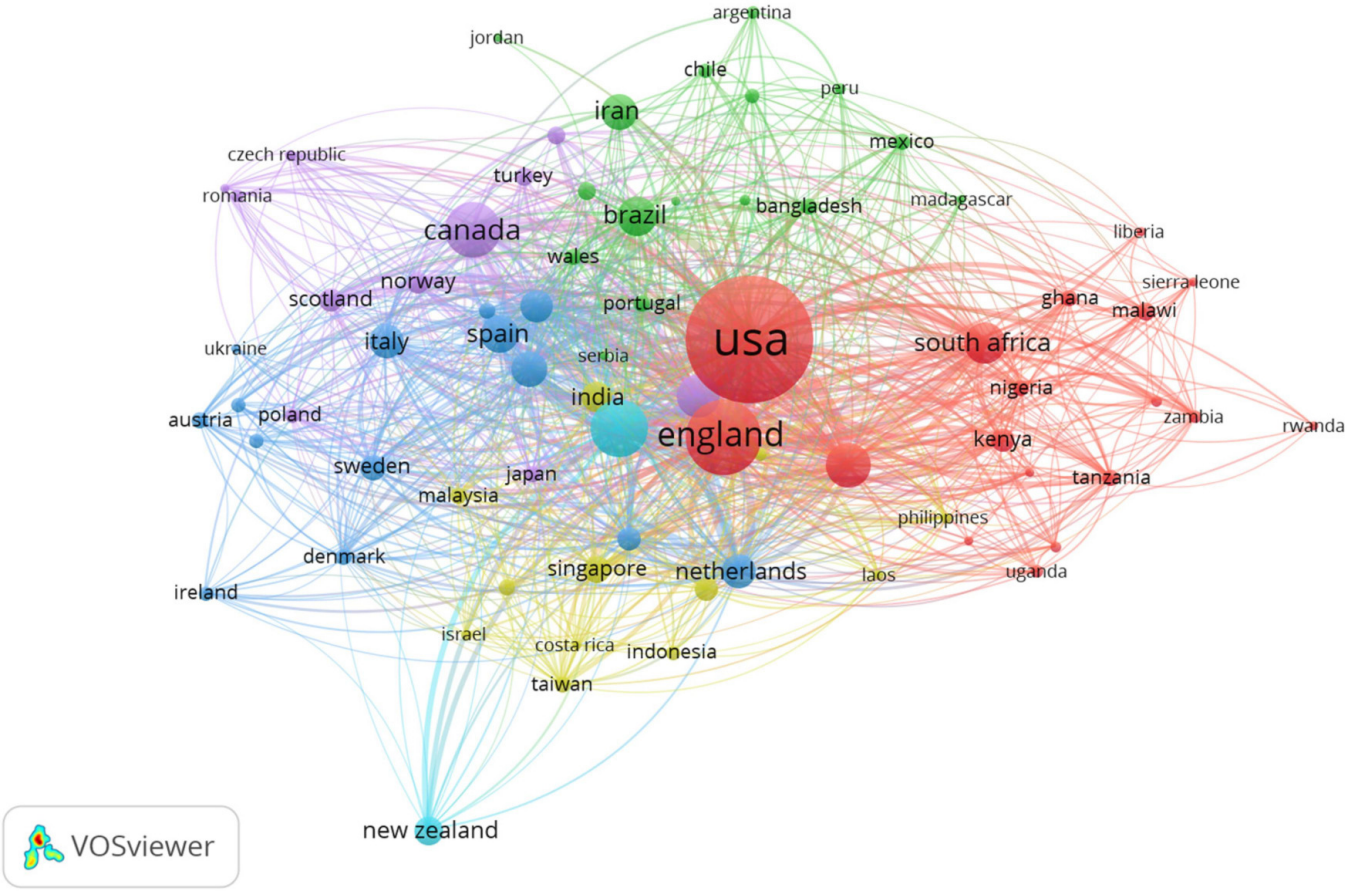
Country bibliographic coupling network.

Figure 13 reveals how countries converge around similar bibliographic sources, suggesting thematic affinity and shared intellectual priorities.

Figure 13 reveals how countries converge around similar bibliographic sources, suggesting thematic affinity and shared intellectual priorities. As expected, the United States, the United Kingdom, and Australia dominate. However, within tightly coupled nodes, countries like Iran and Brazil reflect their growing alignment with global research themes. Importantly, this analysis complements co-authorship and citation maps by showing how national research agendas are intellectually positioned, offering insight into global knowledge integration.

**Figure 14:**
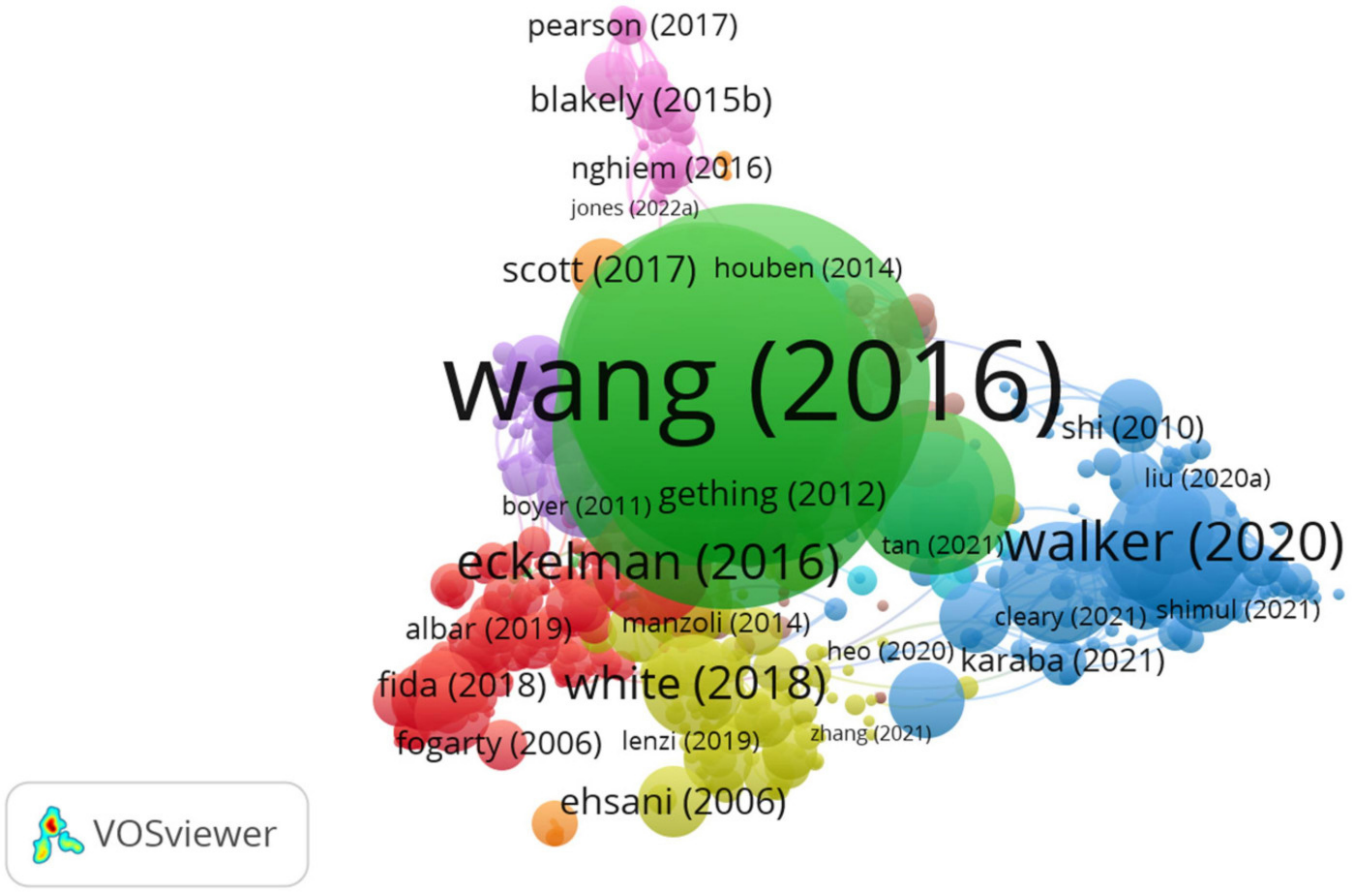
Author bibliographic coupling network.

The dense nodes in Figure 14 represent authors such as Wang, Eckelman, White, and Walker, indicating their strong influence through frequent co-citation. This reflects their foundational roles in advancing interdisciplinary research and methodological innovations in health systems modeling and has made significant contributions to health systems modeling.

## 5 Discussion

The bibliometric analysis identified significant trends and developments in health systems modeling, highlighting contributions from top journals like The Lancet and PLOS One, leading authors such as Blakely T. and Hay S.I., major institutions like the Tehran University of Medical Sciences and the University of Washington, and leading countries like the United States and the United Kingdom. Emerging themes include “health systems”, “modeling”, “predictive modeling”, and “systems dynamics”, suggesting promising directions for future research. The past three decades have seen a sharp rise in the rate of publications in health systems modeling. The number of publications increased significantly from 2015–2023, with 1,606, almost 80% of all the publications in health systems modeling identified in the study. This increase supports the impression that the need to solve complex health issues has warranted new models to support decision-making (83). The annual growth rate of publications is 7.53%, with an average of 9.35 co-authors per article and 37.67% international co-authorship. The most cited article, with 2,881 citations, is by (50), published in The Lancet. The study underscores the importance of epidemiology and predictive modeling, recommending further interdisciplinary collaboration and broadening database integration. Systems modeling is common in diverse health areas, including, amongst many others, disease treatment and management, cancer, mental disease, social media healthcare, emergency surveillance, alcohol and drug use, exercise, food and weight (84).

The quality and reputation of the journals identified in this study testify to the relevance of health systems modeling. Top journals like The Lancet, PLOS One, BMC Public Health, the BMJ, and the Journal of Health Sciences, among others, have a considerable presence in publishing in this research area. The various journals are also receiving much recognition, judging by the h-indexes and total citations of the analyzed articles in each journal in the period under review. Journal articles are the primary means of disseminating scientific information and discoveries (61).

As expected, it can be observed that many authors were from the USA, a country with the highest number of published articles, which has contributed immensely to health systems modeling. This is consistent with (79), who indicated that the USA competes with no one as they are ranked top in scientific production. The two types of articles analyzed were single-country publications (SCP) with all authors from the same country and multiple-country publications (MCP), demonstrating collaboration among authors across countries. Other top authors (albeit far behind the USA) originate from the UK, Australia, Canada, and China. This is in tandem with (64), who used bibliometric analysis for brain-related studies and found that the USA, Canada, the UK, and Australia were among the top-cited countries. The fact that the USA has several institutions that conduct research in health systems modeling and has many prolific authors on the subject matter means that it would also receive many citations.

Another pertinent observation is the confirmation that neither the total number of citations nor the total number of publications can independently provide an author with a good h-index. The h-index, the primary metric to rank scholars, considers the author's total number of articles and citations on the articles (85). This can be illustrated by the fact that Blakely T. has the highest h-index of 15 from 26 articles, despite having fewer total citations (609), compared to Gupta R., who had 17,599 citations from 12 articles but with an h-index of 11.

The USA leads in authors and published journals. As a country in health systems modeling, a surprising observation is that the Tehran University of Medical Sciences in Iran has the highest number of publications. Inferring from the observations in (49), it can be noted that Iran is an emergent economy (almost similar to China) that has contributed considerably to emerging technologies' discourse, like AI. It also shows its prominence in health systems modeling as depicted in Table 9. As expected, institutions from the USA and the UK advocate for more research in health systems modeling, based on Figure 2, Tables 7, 9, 10.

The need to reduce health-related risks, reduce the mortality rate, increase care for humankind, and develop cost-effective outcomes in preventing various health-related diseases were bound to appear as emerging themes from the bibliometric analysis. COVID-19 also appeared as a theme because it is a recent pandemic, prompting scholars to invest much attention in modeling as a step towards making informed decisions about the pandemic (38). In fact, the most focused use of scientific resources may have occurred during the COVID-19 era (61).

Health systems, systems dynamics, and modeling are key themes that have matured, and much research has been invested in these themes. It can also be observed that “health systems” appear in three of the four quadrants of the thematic map depicted in Figure 6. This means that (a) it has matured, (b) it is still growing, although there remains a need for considerable investment, and (c) it is now developed, although the theme is not yet interconnected with other themes in the field.

In addition to revealing scholarly trends, the findings from this bibliometric analysis align with key global policy frameworks such as the WHO Health System Building Blocks (86). The WHO framework identifies six critical components of a well-functioning health system: service delivery, health workforce, information systems, access to essential medicines, financing, and leadership/governance. The findings demonstrate that modeling research increasingly engages with several of these components, particularly health information systems, financing (through cost-effectiveness analysis), and service delivery optimization, illustrating how modeling is used to inform strategic decisions within and across these domains. The prominence of predictive modeling, COVID-19 surveillance, and cost-effectiveness analysis directly supports priorities in pandemic preparedness and response articulated by the WHO Health Emergency Preparedness Framework (87). Moreover, the integration of modeling into domains such as epidemiology and system dynamics reflects broader policy goals around health systems resilience, enabling real-time forecasting, scenario planning, and adaptive decision-making during health shocks (88)These findings position modeling not only as a scientific tool but also as a policy instrument critical for strengthening national and global health system capacities.

### 5.1 Research agenda for health systems modeling

Drawing insights from the themes that emerged from this bibliometric analysis, we propose three priority areas for future research. These priorities are designed to leverage emerging trends and contribute to developing more efficient and effective health systems.

#### 5.1.1 Personalised and precision medicine

As healthcare transitions toward personalized and precision medicine, there is a growing emphasis on developing predictive models that inform individualized treatment strategies. These models integrate diverse data sources such as genomics, clinical history, environmental exposure, and behavioral factors to estimate disease susceptibility, treatment responses, and health trajectories (89–92).

However, despite notable advances, several limitations hinder their real-world application. One major limitation is the lack of data harmonization and interoperability, which complicates the integration of heterogeneous datasets from multiple sources. For instance, electronic health records (EHRs), genomic databases, and lifestyle information are often stored in different formats, making comprehensive modeling challenging (93).

Additionally, bias and underrepresentation in data remain significant obstacles. Many precision medicine models are developed using datasets from high-income countries or specific ethnic groups, reducing their generalizability and increasing the risk of health inequities when applied globally (94).

The explainability and interpretability of AI-driven models also pose challenges for clinical adoption. Clinicians often struggle to trust complex black-box algorithms that do not provide transparent decision-making logic (95).

Future research must address these limitations by:

• Developing standardized data governance frameworks to support multimodal data integration.
• Enhancing representativeness by including diverse populations in model training;
• Prioritizing model transparency and interpretability through explainable AI techniques;
• Conducting clinical validations and impact assessments across real-world healthcare settings.

#### 5.1.2 Modeling for health system resilience to global health threats

The COVID-19 pandemic has shown the need for enhanced models to guide health system responses to large-scale crises such as pandemics and climate-induced disasters. While epidemic modeling has progressed, key limitations constrain the effectiveness of these tools for real-time decision-making.

Many existing models are limited in scope, focusing primarily on disease transmission dynamics [e.g., Susceptible-Infectious-Recovered (SIR) models] while neglecting the operational realities of healthcare delivery, such as workforce capacity, Intensive Care Unit (ICU) availability, supply chain dependencies, and socio-behavioural responses (96). As a result, these models may fail to capture cascading system failures under stress.

Moreover, real-time data scarcity and lag have posed significant constraints. During the early phases of COVID-19, many health systems lacked access to timely and granular data on hospital utilization, stockpile levels, and human resource deployment, data that are critical for dynamic modeling (97).

Another limitation lies in the lack of multi-sectoral integration. Most health system models do not account for interdependencies with sectors such as transportation, education, and the economy, which play crucial roles in crisis response and resource prioritization (98).

To enhance preparedness, future modeling efforts should:

• Adopt systems-thinking approaches that incorporate interdependencies between clinical care, logistics, governance, and public behaviour;
• Integrate real-time data streams for dynamic and adaptive forecasting;
• Design scenario-based simulation tools to test various policy options and their trade-offs;
• Ensure stakeholder engagement in model development to improve relevance and uptake

#### 5.1.3 Interdisciplinary collaboration and stakeholder engagement

Advancing health systems modeling requires interdisciplinary collaboration and stakeholder engagement. Future research should foster collaborations between modelers, clinicians, public health professionals, policymakers, and patients. Engaging stakeholders throughout the research process can ensure that models are relevant, actionable, and aligned with the health system's needs.

#### 5.1.4 Understanding drivers of international research collaboration

While the bibliometric analysis captured inter-country and intra-country collaboration patterns, the underlying motivations or strategic factors influencing why specific countries engage (or do not engage) in collaborations were not examined. These drivers, such as shared health priorities, funding incentives, geographic proximity, historical ties, or policy-driven research agendas, would provide valuable insights into building stronger, more targeted international collaborations in health systems modeling. Therefore, exploring the reasons behind country-level participation in research collaboration represents another important direction for future research.

### 5.2 Contribution of the study

From a practical point of view, this study offers valuable insights for researchers, policymakers, and practitioners in the health sector. In outlining the most prominent scholars, journals, and institutions in health systems modeling, it guides individuals seeking collaboration, publication venues, and authoritative sources. The study's focus on the application of modeling in addressing various health issues, including pandemic response and chronic disease management, underlines the practical utility of modeling techniques in real-world health decision-making. Furthermore, identifying emerging themes and the thematic map of the field can inform funding agencies and research institutions about potential areas for investment and development, thus driving innovation in healthcare solutions and policies.

### 5.3 Limitations of the study

The study relied on data from the WoS database only, which, although extensive, may not capture all relevant publications in the field of health systems modeling. There is an opportunity to expand on the findings from this bibliometric analysis to include other databases to capture a broader spectrum of research outputs in health systems modeling. Another limitation of this study is the absence of consultation with a medical librarian or information retrieval expert to optimize the search strategy. Consequently, there is a possibility of missing relevant literature, especially from non-indexed sources or databases not included in our search. Future studies should aim to employ a more exhaustive search strategy, integrating multiple databases and the expertise of information retrieval specialists to ensure a more comprehensive inclusion of literature.

## 6 Conclusion

In this study, we could ascertain the trajectory, trends, and developments of health systems modeling. The past three decades have seen a sharp rise in the rate of publications in health systems modeling. Scholars are increasingly adding their voices to the discourse on health systems modeling. By analyzing 2,023 articles from various journals and conference proceedings, the study has highlighted the most productive journals, countries, authors, and institutions in health systems modeling. The study has also determined the most frequently used keywords in the research area and their occurrences. It has also provided insight into health systems modeling for a sustainable future using a thematic map. The study acts as a reference point for scholars with an interest in the field of health systems modeling in terms of knowing, for example, the most prominent scholars with whom to collaborate, which journals to publish in, the most active institutions in the research area, and the countries involved in health systems modeling research. Finally, the study provides insights into predictive modeling, especially during pandemics like COVID-19 and epidemiology.

## Data Availability

The raw data supporting the conclusions of this article will be made available by the authors, upon request without undue reservation.
