# Supplementary material for: Trends and developments in health systems modeling: a bibliometric analysis
Source: Front Digit Health. 2025 Oct 14;7:1595310. doi: 10.3389/fdgth.2025.1595310 (PMC12558930; doi:10.3389/fdgth.2025.1595310)
Supplement: Supplementary file 1 [file Supplementaryfile1.pdf]

Supplementary Material: Detailed analysis of the most cited papers

No	Context + Source	Methodology	Findings	Conclusions	Suggestions for Future Research
1	The study aims to provide a comprehensive assessment of all-cause mortality, cause-specific mortality, and life expectancy across the globe from 1980 to 2015. It seeks to inform health policies and interventions by identifying mortality and life expectancy trends. (68)	The study utilised an improved analytical approach, incorporating refinements for estimating child and adult mortality and advancements in data synthesis methods. It expanded the cause list for mortality to include 249 causes. It utilised multiple modeling approaches to assess cause-specific mortality, examining observed and expected levels and trends related to the Socio-demographic Index (SDI).	Global life expectancy increased from 61.7 years in 1980 to 71.8 years in 2015, with substantial gains in sub-Saharan Africa post-2005 due to reductions in HIV/AIDS mortality. However, some regions' life expectancy stagnated or declined due to war, interpersonal violence, or other causes.	The study concludes that despite general progress in life expectancy and mortality reduction, disparities exist, with some countries experiencing declines in life expectancy. It highlights the shifting burden towards non-communicable diseases (NCDs) while noting progress against communicable diseases.	Future research should continue to refine the methodologies for estimating mortality and life expectancy, further investigate the determinants of health disparities, and explore effective strategies for addressing the growing burden of NCDs globally.
2	This study is part of the Global Burden of Disease Study 2016, which comprehensively assesses the prevalence, incidence, and years lived with disability (YLDs) for various diseases and injuries across different regions and countries from 1990 to 2016. (69)	The study uses DisMod-MR 2.1, a Bayesian meta-regression tool. It integrates many data sources, including literature reviews, health surveys, hospital data, and administrative records, to estimate disease and injury incidence, prevalence, and YLDs.	The analysis identifies significant global health challenges, highlighting non-communicable diseases (NCDs) and mental health disorders as leading causes of disability.	The persistent rise in YLDs due to NCDs emphasises the need for global health systems to adapt to the growing demand for chronic disease management and mental health services. The study calls for an enhanced policy focus on preventing and managing NCDs and improving mental healthcare.	Future studies are encouraged to refine disease burden estimates further, explore the underlying determinants of health trends, and assess the effectiveness of health interventions.
3	The study presents a comprehensive analysis of the burden of cardiovascular diseases (CVDs) worldwide, emphasising their prevalence, deaths, and overall impact from 1990 to 2015. (70)	The study integrates data from a wide array of sources, including vital registration, verbal autopsy, health surveys, and administrative data, to estimate mortality, prevalence, and years lived with disability	Despite improvements in some regions, CVD remains a leading cause of health loss worldwide. The findings indicate significant regional variations in CVD burden, with high-income regions showing declines in age-	The study concludes that CVDs continue to pose a major challenge to global health, with substantial disparities in burden and trends across different regions and income levels.	Future research should explore more detailed mechanisms driving the observed trends, investigate the effectiveness of CVD prevention and treatment strategies across different settings, and examine the

		(YLD) for 10 specific causes of CVD.	standardised CVD death rates. In contrast, low-income regions sometimes displayed little change or even increases.		impact of socio-demographic changes on the burden of CVDs.
4	The study seeks to assess changes in health across different locations to aid in health system performance evaluation, resource allocation, policy development, and program decision-making. (71)	Utilizing the Global Burden of Diseases, Injuries, and Risk Factors Study 2016 data, the researchers calculated DALYs and HALE by sex for 195 countries and territories from 1990 to 2016. The study used a composite indicator of the Socio-demographic Index (SDI) to monitor these changes.	Global HALE at birth has increased, with Singapore having the highest for both genders. Overall, there has been an average increase of 6.24 years in global HALE from 1990 to 2016. The study noted a decrease in communicable, maternal, neonatal, and nutritional disease DALYs offset by an increase in non-communicable disease (NCDs) DALYs.	The study concludes that while global DALYs and HALE continue to improve, there is an increase in functional health loss among many populations. Rising Socio-demographic Index (SDI) is associated with increased years lived with disability, a decrease in communicable disease DALYs, and an increase in NCD DALYs.	The authors suggest further investigation into country-specific drivers of disease burden, especially for causes with higher-than-expected DALYs, to inform health policies and targeted prevention efforts.
5	The study addresses the challenge of measuring racial discrimination in health research due to the lack of validated instruments that can be used in large-scale studies. The research seeks to fill this gap by evaluating the "Experiences of Discrimination" (EOD) measure, originally used in the Coronary Artery Risk Development in Young Adults (CARDIA) study. (77)	The study involved participants from a cohort of working-class adults aged 25–64 in Greater Boston, Massachusetts. It included 159 Black, 249 Latino, and 208 White participants in the main study, with additional participants in a validation study.	The EOD measure demonstrated high reliability and validity in capturing self-reported experiences of racial discrimination among working-class African Americans and Latino Americans. It outperformed several single-item discrimination measures in reliability and was significantly associated with psychological distress and, to some extent, cigarette smoking among Blacks and Latinos. The study also highlighted the importance of multi-item measures in racial discrimination research.	The study concludes that the EOD measure is a valid and reliable measure for assessing racial discrimination in population health research. It emphasises the need for such validated instruments to advance the understanding of racism's impact on health.	Future research should focus on the development and validation of measures for other dimensions of racial discrimination, the exploration of discrimination's health impacts across different racial/ethnic groups, and the investigation of the mechanisms through which racial discrimination influences health outcomes.
6	The study highlights the significant impact of the COVID-19 pandemic on	Through a systematic review and meta-regression analysis, the study quantified changes in	The analysis estimated an additional 53.2 million cases of major depressive disorders	The study underscores the urgent need for strengthening mental health systems	Further research to explore and mitigate the indirect effects of the COVID-19

	mental health worldwide, particularly the increase in depressive and anxiety disorders. (74)	the prevalence of major depressive disorders and anxiety disorders during the pandemic.	and 76.2 million cases of anxiety disorders globally due to the pandemic.	worldwide. It suggests incorporating strategies to promote mental well-being and specifically address the determinants of poor mental health exacerbated by the pandemic.	pandemic on mental health is called for. A focus on strategies that can be integrated into public health responses to manage and reduce the growing mental health burden is suggested.
7	The study addresses the gap in understanding the unmet supportive care needs of cancer patients undergoing treatment at public treatment centres in New South Wales (NSW), Australia. (72)	The study used a survey-based approach, involving 1492 consecutive patients from nine major public cancer treatment centres in NSW. The Supportive Care Needs Survey (SCNS) was utilized to cover five areas of need: psychological, health system and information, physical and daily living, patient care and support, and sexuality.	High unmet needs were reported across the five domains, particularly in psychological, health system, information, and physical and daily living areas. Logistic regression modeling identified specific subgroups of patients with varying needs, highlighting the significant predictors of unmet needs according to different domains.	The study concludes that cancer patients experience significant unmet needs in various domains of supportive care. It emphasises the importance of identifying and addressing these needs through targeted interventions to improve the quality of life for individuals living with cancer.	The study suggests further investigation into effective interventions that could address the identified unmet needs of cancer patients. It recommends routine patient needs monitoring as part of oncology care to facilitate the continuous improvement of health services and healthcare professional practices tailored to patient requirements.
8	The study addresses concerns about the indirect effects of the COVID-19 pandemic on maternal and under-5 child mortality, especially in low and middle-income countries (LMICs). (75)	Using the Lives Saved Tool (LiST), the study models three scenarios reflecting varying levels of health system disruption and decreased access to food, predicting additional maternal and under-5 child deaths.	The least severe scenario over six months could result in 253,500 additional child deaths and 12,200 additional maternal deaths. In contrast, the most severe scenario could see 1,157,000 additional child deaths and 56,700 additional maternal deaths.	While recognising the hypothetical nature of its scenarios, the study concludes that the indirect effects of the COVID-19 pandemic could lead to a devastating increase in child and maternal deaths due to disrupted healthcare services and decreased food access.	Future research should explore the impacts of COVID-19 on health system disruptions and food access in LMICs, focusing on developing strategies to mitigate these indirect effects.
9	The study addresses the lack of global estimates for Sickle haemoglobin (HbS). (73)	The study compiled a global database of sickle haemoglobin (HbS) allele frequency surveys and created a map of HbS allele frequency distribution using a Bayesian geostatistical model.	The study revealed high allele frequencies across sub-Saharan Africa, the Middle East, and India, and detailed gene flow in migrations to Western Europe and the eastern coast of the Americas. Estimates indicated that in 2010, approximately 312,000	The study concludes that the impact of HbS on global public health is significant and growing. It emphasises the need for countries and the international community to prioritise diagnoses, genetic counselling, and interventions	Future research directions include improving data collection and analysis methods to assess the HbS allele's distribution and health impact accurately.

			homozygous (SS) neonates and 5,476,000 heterozygous (AS) neonates were born globally, which is higher than previous estimates.	to mitigate the burden of sickle cell disease.	
10	The study identifies gaps in existing methods for evaluating the thermal environment, particularly in their ability to reflect the complexity of human thermoregulation and environmental interactions. It underscores the need for a more sophisticated index that can be universally applied across different climates, regions, and seasons. (76)	The development of the Universal Thermal Climate Index (UTCI) involved a multidisciplinary effort, integrating advanced models of human thermoregulation with environmental data.	The UTCI is presented as a significant advancement in the assessment of the thermal environment, capable of accurately simulating the human body's dynamic response to a wide range of climatic conditions. It overcomes the limitations of previous indices by incorporating comprehensive heat exchange mechanisms and reflecting the physiological impact of temperature, wind, humidity, and radiation.	The authors conclude that the UTCI represents a major step forward in human biometeorology, offering a universal standard for assessing thermal environments. It is poised to enhance applications in public health, urban planning, tourism, and climate impact research by providing a scientifically valid, universally applicable measure of thermal stress or comfort.	The study suggests ongoing refinement and validation of the UTCI, including its adaptation for specific applications and exploring its implications in various fields of study.
